# STING suppresses bone cancer pain via immune and neuronal modulation

**DOI:** 10.1038/s41467-021-24867-2

**Published:** 2021-07-27

**Authors:** Kaiyuan Wang, Christopher R. Donnelly, Changyu Jiang, Yihan Liao, Xin Luo, Xueshu Tao, Sangsu Bang, Aidan McGinnis, Michael Lee, Matthew J. Hilton, Ru-Rong Ji

**Affiliations:** 1 Center for Translational Pain Medicine, Department of Anesthesiology, Duke University Medical Center, Durham, NC USA; 2 Department of Pharmacology and Cancer Biology, Duke University Medical Center, Durham, NC USA; 3 Department of Orthopedic Surgery, Duke University Medical Center, Durham, NC USA; 4 Department of Cell Biology, Duke University Medical Center, Durham, NC USA; 5 Department of Neurobiology, Duke University Medical Center, Durham, NC USA

**Keywords:** Bone cancer, Sensory processing, Experimental models of disease

## Abstract

Patients with advanced stage cancers frequently suffer from severe pain as a result of bone metastasis and bone destruction, for which there is no efficacious treatment. Here, using multiple mouse models of bone cancer, we report that agonists of the immune regulator STING (stimulator of interferon genes) confer remarkable protection against cancer pain, bone destruction, and local tumor burden. Repeated systemic administration of STING agonists robustly attenuates bone cancer-induced pain and improves locomotor function. Interestingly, STING agonists produce acute pain relief through direct neuronal modulation. Additionally, STING agonists protect against local bone destruction and reduce local tumor burden through modulation of osteoclast and immune cell function in the tumor microenvironment, providing long-term cancer pain relief. Finally, these in vivo effects are dependent on host-intrinsic STING and IFN-I signaling. Overall, STING activation provides unique advantages in controlling bone cancer pain through distinct and synergistic actions on nociceptors, immune cells, and osteoclasts.

## Abstract (web summary)

There is an unmet clinical need to develop therapies to alleviate metastatic bone pain, frequently observed in patients with advanced cancers. Here, using mouse models of bone cancer pain, the authors show that STING agonists not only suppress bone cancer tumor burden, but also attenuate bone pain and reduce cancer-induced bone destruction.

## Introduction

Patients with advanced lung, breast, thyroid, and bladder cancers frequently suffer from cancer pain following bone metastasis, which is accompanied by osteolytic lesions and severe pain^1,2^. Studies estimate that ~75% of patients with late stage cancer experience moderate or severe pain^3–5^, and more than half of all patients with metastatic cancer pain report insufficient pain relief by the currently available pharmacotherapies^6,7^. Inadequate pain control for patients with metastatic cancer is often accompanied by depression, anxiety, impaired function, and significantly reduced quality of life, leading to increased morbidity and mortality^8–10^. Thus, in addition to the ongoing challenge of developing new therapeutics capable of treating the underlying cancer, there is also an urgent unmet clinical need to develop new therapies, which provide cancer patients with palliative care to relieve pain and improve quality of life. Notably, endocannabinoid has been shown to alleviate murine bone cancer pain without producing the side effects of opioids^11^. An ideal therapeutic approach would be one that is capable of actively treating the underlying cancer while concurrently suppressing cancer-associated pain, thereby treating the pain and the underlying disease.

STING, or stimulator of interferon (IFN) genes, is an intracellular DNA sensor which plays a critical role in innate immunity, promoting the elimination of pathogens and damaged host cells via the induction of type-I IFN (IFN-I), including IFN-α and IFN-β. Activation of the STING pathway can also potently enhance antitumor immunity, underscored by preclinical studies in which the murine STING agonist DMXAA or the cross-species STING agonist ADU-S100 have been demonstrated to suppress tumor progression and increase survival in an adaptive immune cell-dependent manner^12–17^. In addition, several groups have demonstrated STING-activating- micro- or nanoparticles also show efficacy in promoting innate and adaptive immunity in orthotopic and genetically engineered tumor models in mice^18–20^. These studies have led to the exploration of ADU-S100 and other small molecule STING agonists to be tested as potential immunotherapy agents in several ongoing clinical trials^21,22^. Our recent study has shown that STING agonists could effectively control nociception in naïve animals and animals with pathological pain^23^. However, it remains unclear if STING agonists are effective in treating bone cancer, especially given that bone marrow is regarded to be an immunosuppressive tumor microenvironment^24^.

Previous studies have demonstrated that IFN-I signaling suppresses osteoclast formation, and activation of the STING pathway promotes bone formation in a murine bone autoimmune disease model^25–28^. This is noteworthy, as tumor-induced over-activation of osteoclasts is a dominant mechanism leading to osteolytic bone lesions and cancer pain^29^. It is generally believed that activation of pain-sensing nociceptive neurons (nociceptors) by soluble mediators released from cancer cells and osteoclasts drives bone cancer pain. Based on these studies, and taken in conjunction with the promise STING agonists have shown as cancer immunotherapy agents, we posited that activation of the STING pathway in bone cancer may be a unique synergistic approach to concurrently promote antitumor immunity, suppress bone destruction, and provide pain control.

In this study, we tested the hypothesis that STING agonism would be particularly efficacious in attenuating cancer pain using multiple syngeneic mouse models of bone cancer pain. Thus, in this study we demonstrate that small molecule agonists of STING provide long-lasting relief from cancer-induced bone pain via distinct and synergistic actions on pain-sensing nociceptors, adaptive immune cells, and osteoclasts through a mechanism dependent on IFN-I signaling.

## Results

### STING agonists attenuate bone cancer-induced pain and restore locomotor function

**Fig. 1:**
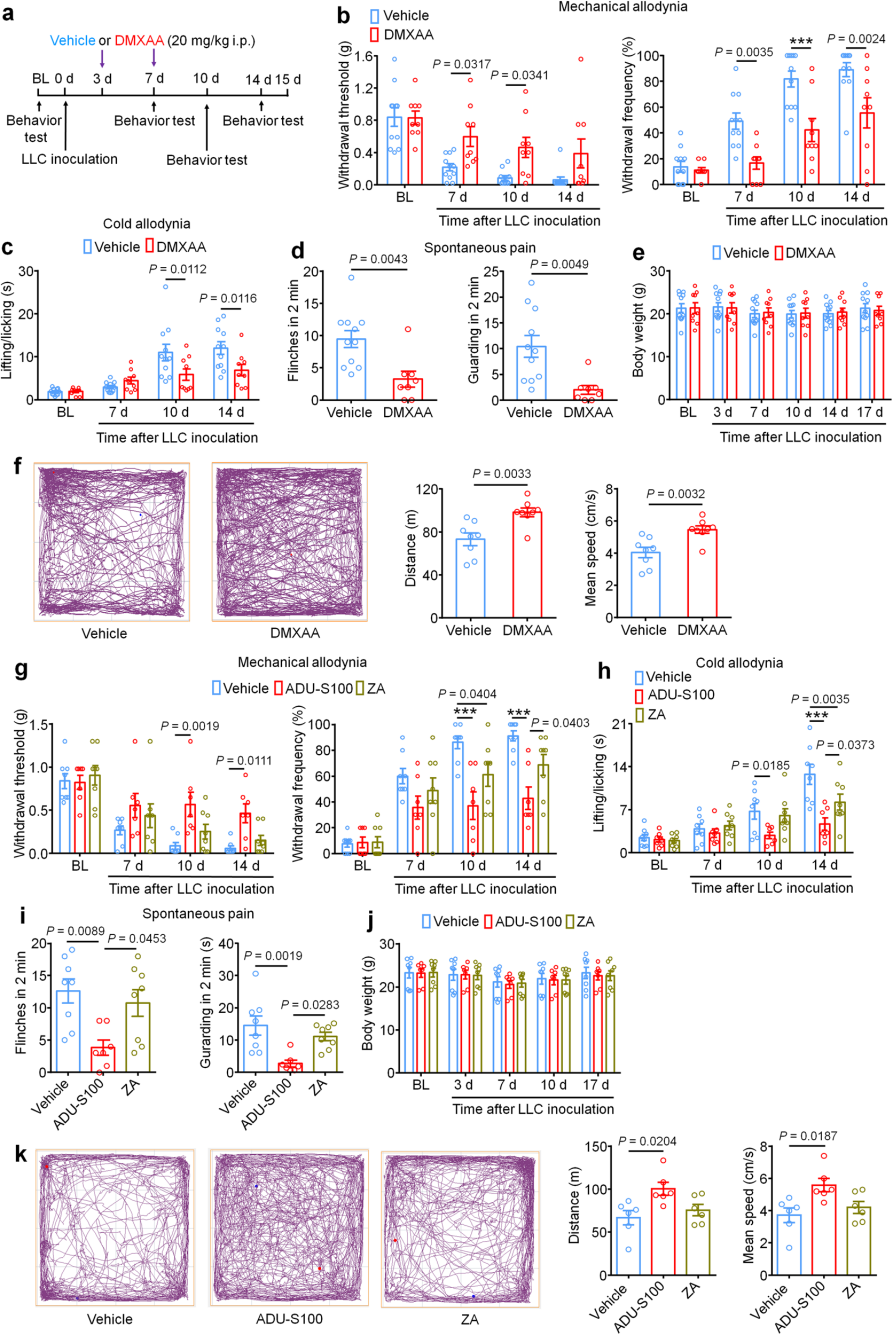
STING agonists reduce bone cancer-induced pain and functional impairment. **a** Experimental design to test the antinociceptive effects of DMXAA in the LLC model of bone cancer. **b** Von Frey testing to determine cancer-induced mechanical allodynia, as assessed by withdrawal threshold (left) or withdrawal frequency (0.16 g stimulus; right) in mice treated with vehicle or DMXAA (20 mg/kg, i.p.) (*n* = 11 vehicle-treated mice and *n* = 9 DMXAA-treated mice) ***P < 0.001. **c** Assessment of cancer-induced cold allodynia after LLC inoculation in mice treated with vehicle or DMXAA (*n* = 11 vehicle-treated mice and *n* = 9 DMXAA-treated mice). **d** Comparison of spontaneous pain as indicated by flinching behaviors (left) or guarding behaviors (right) in vehicle and DMXAA-treated mice on d14 after tumor implantation (*n* = 11 vehicle-treated mice and *n* = 8 DMXAA-treated mice). **e** Measurement of body weight in vehicle or DMXAA-treated mice at the indicated timepoints (*n* = 11 vehicle-treated mice and *n* = 9 DMXAA-treated mice). **f** Open field testing at d14 post-inoculation to determine distance traveled (m) and mean speed (cm/s) over a 30 min duration in vehicle or DMXAA treated mice (*n* = 8 mice/group). Left: representative traces. Right: quantification. **g** Von Frey testing to measure cancer-induced mechanical allodynia in mice treated with vehicle, ADU-S100 (20 mg/kg, i.p.) or ZA (zoledronic acid; 100 µg/kg, i.p.), *n* = 8 vehicle-treated mice and *n* = 7 ADU-S100-treated mice, and *n* = 8 ZA-treated mice, ***P < 0.001. **h** Measurement of cancer-induced cold allodynia in mice with indicated treatment on day 7, 10 and 14 after LLC inoculation (*n* = 8 vehicle-treated mice and *n* = 7 ADU-S100-treated mice, and *n* = 8 ZA-treated mice). **i** Spontaneous pain as determined by flinching behaviors (left) or guarding behaviors (right) over a 2-min interval on d14 post inoculation (*n* = 8 vehicle-treated mice and *n* = 7 ADU-S100-treated mice, and *n* = 8 ZA-treated mice). **j** Body weight measurements in mice with the indicated treatments (*n* = 8 vehicle-treated mice and *n* = 7 ADU-S100-treated mice, and *n* = 8 ZA-treated mice). **k** Open field testing, measuring distance traveled (m) and mean speed (cm/s) over a 30 min duration in vehicle, ADU-S100, and ZA-treated mice at d14 after tumor inoculation (*n* = 6 mice/group). Left: representative traces; right: quantification. All data displayed represent the mean ± SEM, repeated-measures two-way ANOVA with Bonferroni’s post-hoc test (**b**, **c**, **e**, **g**, **h**, **j**); two-tailed Student’s *t*-test (**d**, **f**); one-way ANOVA with Bonferroni’s *post-hoc* test (**i**, **k**). Source data are provided as a Source Data file.

We first sought to determine whether activation of STING via systemic administration of DMXAA could provide long-term therapeutic effects in a mouse model of metastatic bone cancer. To this end, we established a syngeneic murine bone cancer pain model by inoculating Lewis lung carcinoma (LLC, 2 × 10^5^ cells in 2 µl) cells into the femora of C57BL/6 mice. Vehicle or DMXAA (20 mg/kg) was intraperitoneally (i.p.) injected twice to these mice on day 3 (3d) and day 7 (7d) after tumor implantation. Behavioral tests including von Frey testing for mechanical allodynia and acetone response duration for cold allodynia were performed on the hindpaw of the tumor-bearing leg at baseline (BL), 7d (before drug injection), 10d, and 14d after LLC inoculation. Flinches and guarding behavior for spontaneous/ongoing pain was evaluated on day 14 post tumor injection (Fig. 1a), as spontaneous pain behaviors were infrequently observed at earlier time points in this model. DMXAA treatment significantly reduced mechanical allodynia on d7, d10, and d14 (Fig. 1b) and cold allodynia on d10 and d14 after LLC implantation (Fig. 1c). On d14, DMXAA treatment also attenuated measures of spontaneous and ongoing pain (Fig. 1d). No apparent sex differences were observed, as the therapeutic effect of DMXAA on bone cancer pain existed in both male and female mice (Supplementary Fig. 1a–d), which is congruent with our previous report^23^. In addition, we observed no differences in body weight between the vehicle- and DMXAA-treated groups (Fig. 1e), indicating the experimental protocol we used is relatively safe and without gross systemic gastrointestinal (GI) toxicity. Notably, survival was not the endpoint of this study and given that animals in late stages of this model experienced severe pain and functional impairment, all mice were sacrificed at d17 post-inoculation to maintain reasonable health conditions and minimize suffering, as in our recent study^30^.

Clinically, a critical comorbidity of bone metastasis in patients with advanced stage cancers is diminished or lost mobility, leading to functional impairment and reduced quality of life^2,8^. To determine whether STING activation with DMXAA could improve locomotor function, we evaluated the movement activity in the open field test. Importantly, mice treated with DMXAA exhibited greater overall distance of movement and increased speed of movement on d14 after tumor inoculation (Fig. 1f). Thus, systemic treatment with DMXAA significantly improved locomotor function in mice with bone cancer.

Bisphosphonates are widely used for the prevention and treatment of metastatic bone cancer-induced skeletal-related events (SREs) by promoting apoptosis of bone-resorbing osteoclasts. Zoledronic acid (ZA) is one of the most potent bisphosphonates^31^, and has also been reported to exhibit antitumor effects^32^. Moreover, given the limited translational significance of DMXAA due to its specificity for murine STING, we also sought to test whether ADU-S100 could exert similar therapeutic effects. Following administration of vehicle, ADU-S100 (20 mg/kg), or ZA (100 µg/kg, a highly effective dose with minimal toxicity, as demonstrated in previous studies^31,32^ at d3 and d10, we found that ZA failed to reduce cancer-induced mechanical allodynia when analyzing paw withdrawal threshold, but could reduce withdrawal frequency to low-threshold stimulation (0.16 g Von Frey filament) on d10 post tumor implantation. ADU-S100 treatment, however, could significantly attenuate mechanical allodynia in both measures on d10 and d14, with effects superior to those of ZA (Fig. 1g). ZA reduced cold allodynia on d14 after tumor inoculation, whereas ADU-S100 reduced cold allodynia on both d10 and d14, and this effect was significantly greater in the ADU-S100 group compared to the ZA group on d14 (Fig. 1h). In addition, mice treated with ADU-S100 but not ZA exhibited reduced spontaneous and ongoing pain compared to vehicle-treated mice (Fig. 1i). We found that these doses of ADU-S100 and ZA again had no effect on overall body weight (Fig. 1j), suggesting they are relatively safe and without gross GI toxicity. To again assess the potential benefits of ZA and ADU-S100 on function and mobility, we performed open field testing at d14 on mice treated with vehicle, ADU-S100 and ZA at d3 and d10. Notably, mice in the ADU-S100 treatment group but not the ZA treatment group exhibited increased overall distance of movement and increased speed of movement compared with the vehicle treatment group (Fig. 1k). Taken together, ADU-S100 was superior to ZA in reducing cancer-induced pain and improving locomotor function.

### STING agonists protect against cancer-induced bone destruction

**Fig. 2:**
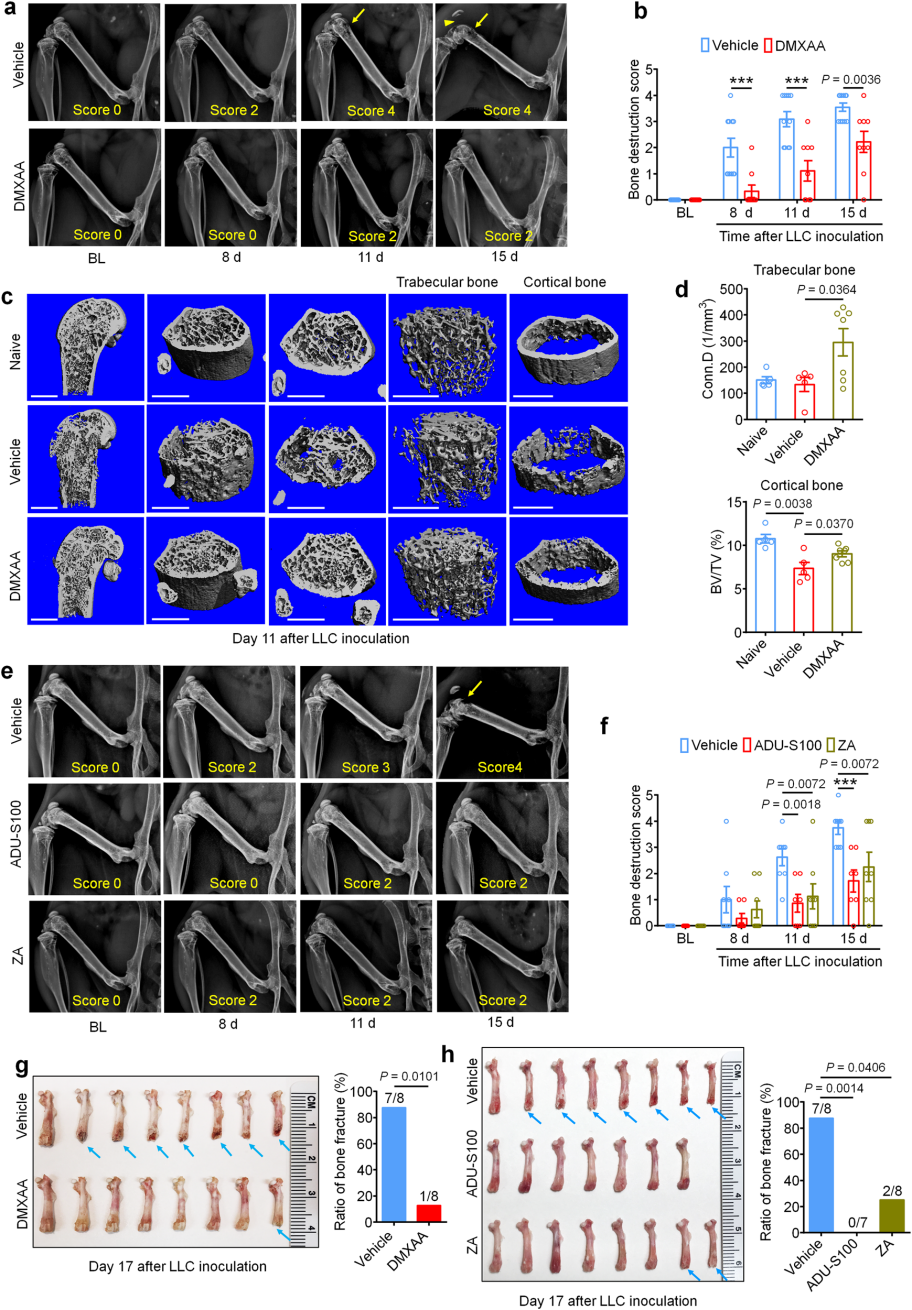
STING agonists attenuate cancer-induced bone destruction. **a** Representative radiographs of tumor-bearing femora from vehicle or DMXAA treated mice. Bone destruction score is indicated in each image and arrows show bone lesions with scores over 3 while arrowhead shows the detached patella. **b** Quantification for (**a**) (*n* = 11 vehicle-treated mice and *n* = 9 DMXAA-treated mice), ***P < 0.001. **c** Micro-CT images showing trabecular and cortical bone destruction in the distal part of tumor-bearing femora on d11 after LLC inoculation. Scale bars, 1 mm. **d** Morphometric quantification of micro-CT images with analysis of trabecular bone (Conn.D; upper) or cortical bone (BV/TV) in tumor-free femora from naïve mice or LLC inoculated femora from vehicle or DMXAA-treated mice (*n* = 5 naive mice, *n* = 5 vehicle-treated tumor-bearing mice, and *n* = 7 DMXAA-treated tumor-bearing mice). **e**, **f** Radiographical analysis of bone destruction in mice administered vehicle, ADU-S100, or ZA at the indicated timepoints after tumor inoculation. **e** Representative X-ray images. Bone destruction score is labeled on the bottom of each photo and arrow indicates bone destruction score more than 3. **f** Quantification of images in **e** (*n* = 8 vehicle-treated mice and *n* = 7 ADU-S100-treated mice, and *n* = 8 ZA-treated mice), ***P < 0.001. **g** Images of femurs and quantification of the proportion with bone fracture from tumor bearing femora taken from vehicle or DMXAA-treated mice on d17 after LLC inoculation. Arrows indicate the disconnection and absence of the distal part of the femora (*n* = 8 mice/group). **h** Images of tumor bearing femora with indicated treatment harvested on d17 after LLC inoculation (left) and quantification of the proportion with bone fracture (right). Arrows indicate lesion and loss of the distal aspect of the femora (*n* = 8 vehicle-treated mice and *n* = 7 ADU-S100-treated mice, and *n* = 8 ZA-treated mice). All data indicate the mean ± SEM, repeated-measures two-way ANOVA with Bonferroni’s *post-hoc* test (**b**, **f**); two-tailed Student’s t-test (**d**); two-sided Fisher’s exact test (**g**, **h**). Source data are provided as a Source Data file.

**Fig. 3:**
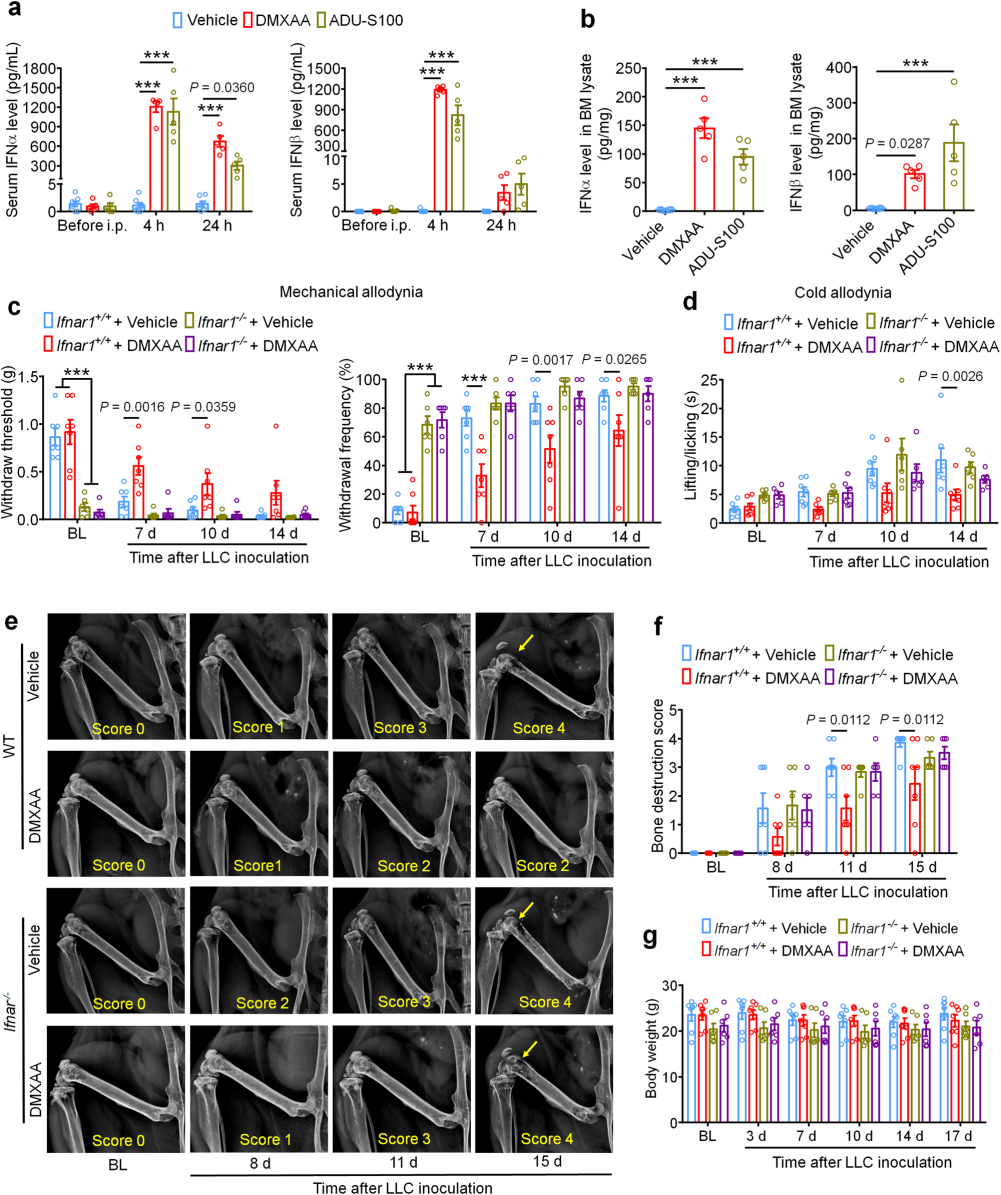
The protective effects of STING agonists are mediated by host IFN-I signaling. **a** Analysis of serum IFN-α (left) and IFN-β (right) levels at BL and again 4 h or 24 h after DMXAA or ADU-S100 treatment on d3 after tumor implantation (*n* = 7 vehicle-treated mice, *n* = 5 DMXAA-treated mice, and *n* = 5 ADU-S100-treated mice), ***P < 0.001. **b** IFN-α (left) and IFN-β (right) levels in bone marrow (BM) lysates from mice treated with vehicle, DMXAA or ADU-S100 (*n* = 8 vehicle-treated mice, *n* = 5 DMXAA-treated mice, and *n* = 5 ADU-S100-treated mice), ***P < 0.001. **c** von Frey testing to determine withdrawal threshold (left) and frequency (right) from *Ifnar1*^*+/+*^ or *Ifnar1*^*−/−*^ mice treated with vehicle or DMXAA (2 x 20 mg/kg, i.p.). **d** Analysis of cold allodynia in vehicle or DMXAA-treated *Ifnar1*^*+/+*^ mice and *Ifnar1*^*−/−*^ mice. **e**–**f** Bone destruction scores from radiographs of tumor bearing femora in *Ifnar1*^*+/+*^ and *Ifnar1*^*−/−*^ mice with the indicated treatment on d0, d8, d11 and d15 after LLC injection. Arrows show bone lesions with destruction scores over 3. **e** Representative X-ray images. **f** Quantification for **e**. **g** Body weight measurement after vehicle or DMXAA treatment. Sample sizes for **c**–**g** were as follows: *n* = 7 vehicle-treated *Ifnar1*^*+/+*^ mice, *n* = 7 DMXAA-treated *Ifnar1*^*+/+*^ mice, *n* = 6 vehicle-treated *Ifnar1*^*−/−*^ mice, *n* = 6 DMXAA-treated *Ifnar1*^*−/−*^ mice. All data indicate the mean ± SEM, repeated-measures two-way ANOVA with Bonferroni’s *post-hoc* test (**a**, **c**, **d**, **f**, **g**); one-way ANOVA with Bonferroni’s *post-hoc* test (**b**). Source data are provided as a Source Data file.

Bone cancer-induced pain usually develops in tandem with the onset of tumor-induced bone destruction. It is understood that bone cancer pain is evoked by factors produced directly by cancer cells which act on afferent nociceptive nerve fibers in the tumor microenvironment (TME)^33,34^. In addition, cancer cells promote bone cancer pain indirectly by accelerating osteoclastogenesis, generating osteoclasts which release pro-nociceptive factors and promote bone resorption, facilitating bone destruction and painful fractures^30,35^. Thus, cancer cells in the bone tumor microenvironment promote bone pain through direct and indirect mechanisms (Supplementary Fig. 1e). In vivo, the LLC cell line is known to induce osteolytic bone destruction due to tumor-induced activation of osteoclast formation and activity^30^, recapitulating the pathogenesis of metastatic osteolytic bone cancers in humans. Thus, we sought to continuously assess bone destruction using radiography in tumor-bearing mice treated with vehicle or DMXAA (20 mg/kg, i.p. at d3 and d7). The grade of bone destruction was scored on a range from 0 to 5 using high-resolution X-ray radiographs of tumor bearing femora, as described by Honore et al.^36^. In this model, we observed that LLC inoculation produces a progressively worsening pattern of bone destruction which is localized to the distal femur while leaving the proximal aspect of the femur relatively unperturbed (Fig. 2a). Moreover, the tendon connecting the femur and patella is frequently severed (arrowhead; Fig. 2a). DMXAA treatment decreased the bone destruction score on d8, d11 and d15 after LLC inoculation compared with vehicle group (Fig. 2a, b). No sex differences were observed in the protective effects of DMXAA on bone destruction (Supplementary Fig. 1f, g). To explore the microarchitecture of bone, we also employed micro computed tomography (Micro-CT) with 3-dimentional reconstruction analysis ex vivo on the distal aspect of tumor-bearing femurs. On d11 after tumor implantation and vehicle or DMXAA treatment as in Fig. 2a and 3D reconstruction showed less bone cancer-induced trabecular bone loss and fewer cortical bone lesions in DMXAA-treated mice compared with vehicle group (Fig. 2c). Notably, at d11 after LLC inoculation, the loss of cortical bone was most apparent when compared to naïve (non-tumor-bearing) mice. Quantitative assessments for bone microstructural parameters demonstrated there is higher trabecular bone connectivity density (Conn.D) relative to both naïve and LLC-bearing, vehicle-treated mice (Fig. 2d). In addition, DMXAA rescued LLC-induced cortical bone loss (as assessed by cortical bone volume/total volume (BV/TV; Fig. 2d). The protective effects of DMXAA on other microstructural parameters of trabecular bone were less obvious, although we did observe a significant increase in the number of trabeculae (Supplementary Figs. 2a–d). Next, we analyzed bone destruction by X-ray radiography following treatment with ADU-S100 or ZA (d3 and d10, as in Fig. 1g). Similar to DMXAA, both ADU-S100 and ZA treatment reduced the bone destruction score at d11 and d15 after tumor inoculation (Fig. 2e, f).

Development and progression of cancer-induced osteolytic bone destruction frequently leads to bone fracture, which is an important component of SREs in patients with bone metastasis and is associated with decreased overall survival^37^. On d17 after LLC inoculation, mice were euthanized and the tumor bearing femora were collected and the distal tumor-bearing femur where bone destruction occurs was analyzed. Notably, we found that 87.5% (7/8 mice) of vehicle-treated mice suffered bone fractures, whereas only 12.5% (1/8 mice) mice treated with DMXAA developed distal bone fractures (Fig. 2g). Likewise, 0% (0/7 mice) in the ADU-S100-treated group and 25% (2/8 mice) in the ZA group developed bone fractures (Fig. 2h), indicating both STING agonists and ZA could significantly reduce bone destruction.

### STING agonist treatment protects against breast cancer induced bone pain and bone destruction

Similarly to lung cancer, breast cancer is also prone to metastasize to bones and cause bone destruction^8^. To explore the potential protective effect of STING agonists in breast cancer-induced bone destruction, we utilized the E0771 medullary breast carcinoma cell line to establish a syngeneic mouse model of breast cancer-induced bone cancer pain in female C57BL/6 mice, as 98% of all breast cancers occur in females^38^. Similar to the LLC line, tumors established with the E0771 line also induce osteolytic bone lesions^39^. After intra-femur inoculation, mice were treated with vehicle, DMXAA or ADU-S100 followed by behavioral testing and X-ray radiography of tumor-bearing femurs (Supplementary Fig. 3a). Similar to our results in the LLC bone cancer pain model, we found that DMXAA and ADU-S100 treatment could markedly reduce mechanical allodynia, cold allodynia and spontaneous pain compared to vehicle treatment (Supplementary Fig. 3b–d) but had no effect on body weight (Supplementary Fig. 3e). Furthermore, both DMXAA and ADU-S100 could also attenuate bone destruction scored from X-ray images of the E0771-bearing femora (Supplementary Fig. 3f, g). Thus, STING agonists can protect against cancer-induced bone pain and bone destruction caused by multiple cancer subtypes prone to bone metastasis.

### Protective effect of DMXAA on bone pain and bone destruction is STING dependent

To verify the antinociceptive and bone anabolic effects of DMXAA are mediated by STING, WT mice and STING “goldenticket” knockout (STING^*gt/gt*^) mice^40^ were inoculated with LLC cells intrafemorally followed by vehicle or DMXAA (20 mg/kg) administration (i.p.) on d3 and d7 post LLC injection. Notably, STING^*gt/gt*^ mice displayed markedly reduced hindpaw withdrawal threshold and increased withdrawal frequency in von Frey tests compared to WT mice at baseline. DMXAA treatment significantly attenuated mechanical and cold allodynia in WT mice, and this effect was abolished in STING^*gt/gt*^ mice (Supplementary Fig. 4a, b). We also measured cancer-induced bone destruction in these mice using radiographic examination of bone destruction of the tumor-bearing distal femurs. We observed a reduction in the bone destruction score in DMXAA-treated WT mice at d11 and d15 after tumor inoculation, and this effect was abolished in STING^*gt/gt*^ mice (Supplementary Fig. 4c, d). We did not see body weight changes after the experimental manipulations (Supplementary Fig. 4e). Thus, as expected, the protective effects of DMXAA on cancer-induced pain and bone destruction are mediated by STING.

### IFN-I signaling mediates the protective effects of STING agonists in bone cancer

STING activation leads to the transcriptional induction of interferon response genes and the robust production and release of type-I interferons, including IFN-α and IFN-β. To confirm that systemic administration of STING agonists leads to increased IFN-I response both systemically and locally within the tumor microenvironment, we analyzed the level of IFN-α and IFN-β by ELISA. We found that serum levels of IFN-α increased ~1000-fold 4 h after a single i.p. injection of DMXAA (20 mg/kg) or ADU-S100 (20 mg/kg) on d3 after tumor inoculation compared to vehicle group, and this increase was maintained for up to 24 h. Meanwhile, serum IFN-β levels were also dramatically upregulated 4 h after DMXAA and ADU-S100 administration (Fig. 3a). On d3 after LLC implantation, the bone marrow (BM) from tumor bearing femora were also collected 4 h after vehicle, DMXAA or ADU-S100 i.p. treatment and analyzed by ELISA. Both IFN-α and IFN-β were sharply increased in BM lysate in mice treated with DMXAA or ADU-S100 (Fig. 3b). Thus, systemic administration of STING agonists promoted a robust IFN-I response systemically and in the bone cancer tumor microenvironment.

The IFN-α/β receptor (IFNAR) is a heterodimeric signal transducing receptor complex composed of Ifnar1 and Ifnar2, each of which is required for IFN-I signaling. To test how IFN-I signaling contributes to the protective effects of STING agonists in the bone cancer model, we again introduced LLC cells into the femora of *Ifnar1*^*+/+*^ (WT) or *Ifnar1*^*−/−*^ (KO) mice to establish the bone cancer models in mice with deficient host IFN-I signaling. Similar to mice lacking STING, we found that *Ifnar1*^*−/−*^ mice exhibited mechanical hypersensitivity at baseline compared to WT littermate control mice (Fig. 3c). Following treatment with vehicle or DMXAA (20 mg/kg, i.p. at d3 and d7), we found that DMXAA treatment effectively attenuated cancer-induced mechanical allodynia and cold allodynia in WT mice but not *Ifnar1*^*−/−*^ mice (Fig. 3c, d). On d11 and d15 after tumor inoculation, DMXAA treatment also significantly reduced the bone destruction score without changing overall body weight in WT mice, but this effect was abolished in *Ifnar1*^*−/−*^ mice (Fig. 3e–g). Therefore, host IFN-I signaling through Ifnar1 is required for the protective effects of STING agonists on cancer pain and bone destruction induced by bone cancer.

### DMXAA inhibits bone cancer-induced hyperexcitability of DRG nociceptive neurons

**Fig. 4:**
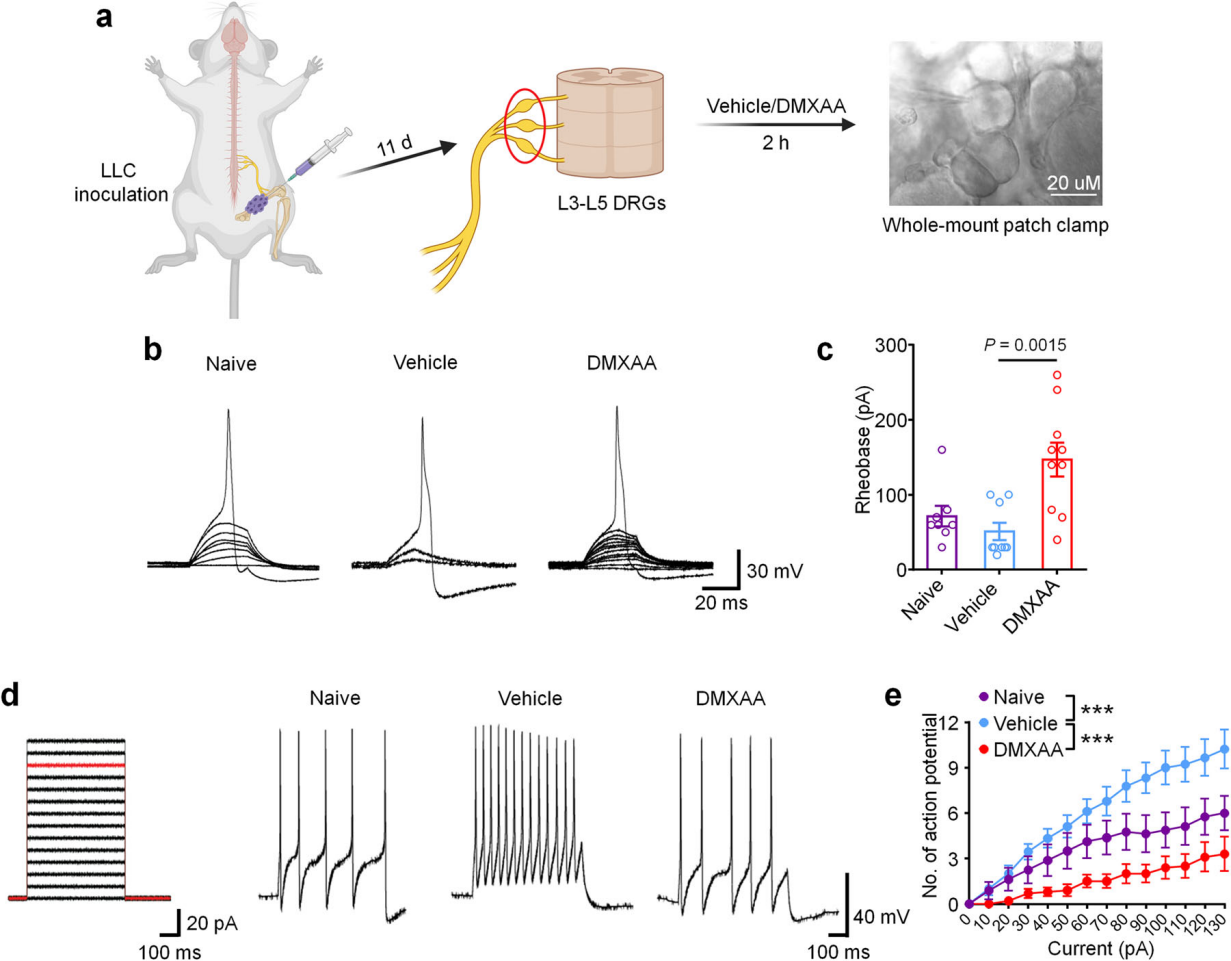
Ex vivo STING activation inhibits neuronal hyperexcitability of DRGs from cancer-bearing mice. **a** Schematic of whole-mount DRG preparation, drug treatment, and bright field image with a recording micropipette sealed on a small-diameter neuron (nociceptor). **b** Representative traces of rheobases: current clamp recordings of the membrane potential from small diameter DRG neurons of naïve mice or bone cancer-bearing animals with or without DMXAA (30 µM, 2 h). Current injection for action potential induction starts from 0 pA and increases 10 pA per step for 30 ms. **c** The averages of rheobases from naïve mice, vehicle or DMXAA-treated group (naïve: *n* = 8 neurons/4 mice; vehicle: *n* = 9 neurons/4 mice; DMXAA: *n* = 10 neurons/4 mice). **d** Left: injected current steps for the induction of action potentials starts from 0 pA and increases 10 pA per step for 300 ms. Right: representative traces showing the response to a 110 pA current injection (red line in left) from small diameter DRG neurons of naïve mice or bone cancer mice with or without DMXAA treatment. **e** Action potentials in response to increasing current amplitude from naïve mice or bone cancer mice with or without DMXAA (naïve: *n* = 8 neurons/4 mice; vehicle: *n* = 9 neurons/4 mice; DMXAA: *n* = 10 neurons/4 mice), ***P < 0.001. All data indicate the mean ± SEM, one-way ANOVA with Bonferroni’s *post hoc* test (**c**); repeated-measures one-way ANOVA with Bonferroni’s *post-hoc* test (**e**). Source data are provided as a Source Data file.

Given that cancer-induced bone pain in our model is transduced by peripheral nociceptors in the dorsal root ganglion (DRG), we next sought to determine whether STING signaling in peripheral sensory neurons contributes to the antinociceptive effects of STING agonists in bone cancer pain. Given our previous report in which we demonstrated that STING agonists can directly suppress nociceptor hyperexcitability in a chemotherapy-induced peripheral neuropathy (CIPN) model of chronic pain^23^, we posited that STING agonists may also acutely attenuate bone cancer-induced hyperexcitability of peripheral nociceptors, To test this hypothesis, WT mice were inoculated with LLC cells to establish bone cancer models and lumbar L3–L5 DRGs were isolated on d11 and incubated ex vivo with vehicle or DMXAA (30 µM) for 2 h followed by patch clamp recordings to measure nociceptor excitability (Fig. 4a). Importantly, in this paradigm, the DRGs from tumor-bearing mice retain their hyperexcitable state relative to DRGs from naïve mice, enabling us to test whether DMXAA-mediated STING activation in DRGs can directly reduce nociceptor excitability. Compared to vehicle, DMXAA incubation of DRGs markedly increased the rheobase of nociceptors, a measure of the current required to evoke action potentials (Fig. 4b, c). In addition, upon examination of current-evoked action potentials, we found that while vehicle-treated tumor-bearing mice exhibited nociceptor hyperexcitability relative to naïve (non-tumor-bearing) mice, DMXAA could completely reverse this cancer-induced nociceptor hyperexcitability (Fig. 4d–e). Although our sample sizes are relatively small given the complexity of this ex vivo electrophysiological recording approach, the particularly large magnitude of the effects enabled us to see statistically significant results. Taken together, these data indicate that STING activation with DMXAA can suppress cancer-induced hyperexcitability of DRG nociceptors. Given that DRGs in this preparation are axotomized, thus divorcing them from peripheral or central cell types that could contribute to excitability changes, we conclude that cells present within the DRG are sufficient to mediate these effects.

**Fig. 5:**
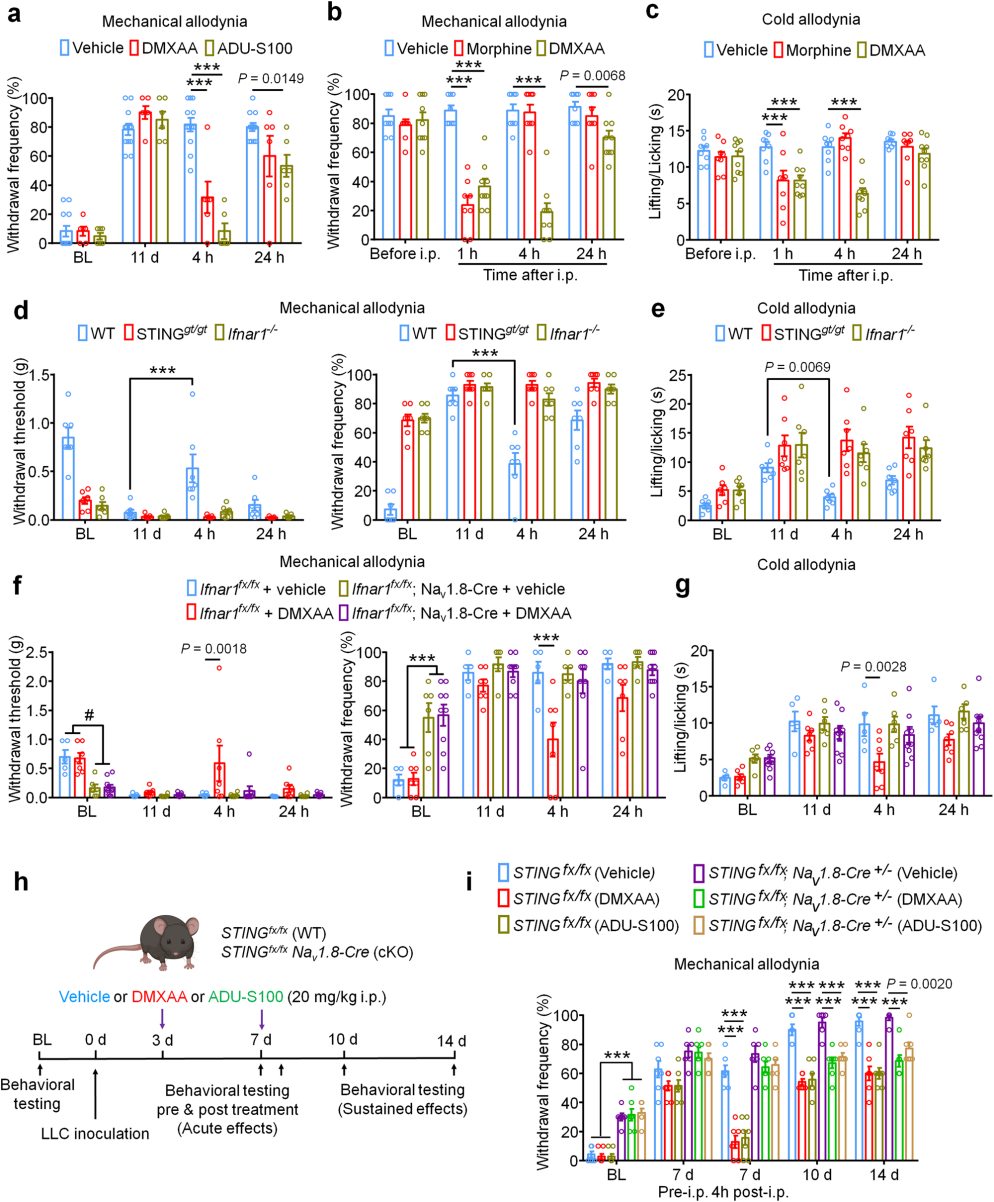
Direct antinociceptive effect of STING agonist is dependent on STING and Ifnar1 in sensory neurons. **a** Von Frey testing to determine cancer-induced mechanical allodynia, as assessed by withdrawal frequency in mice treated with vehicle (*n* = 12 mice), DMXAA (20 mg/kg, i.p., *n* = 6 mice) or ADU-S100 (20 mg/kg, *n* = 6 mice), ***P < 0.001. Measurement of mechanical allodynia as indicated by paw withdrawal frequency (**b**) or cold allodynia (**c**) 1 h, 4 h or 24 h after a single i.p. injection of vehicle (*n* = 8 mice), DMXAA (20 mg/kg, *n* = 9 mice), or morphine (10 mg/kg, *n* = 8 mice) on d11 after LLC inoculation, ***P < 0.001. **d**-**e** Measurement of mechanical allodynia by von Frey testing (**d**) and cold allodynia from acetone response (**e**) after DMXAA i.p. injection in WT, STING^*gt/gt*^ or *Ifnar1*^*−/−*^ mice (*n* = 7 mice/group). Measurement of mechanical allodynia by von Frey testing (**f**) and cold allodynia by acetone response duration (**g**) after vehicle/DMXAA i.p. injection in Ifnar1^fx/fx^; Na_v_1.8-Cre (Ifnar1-cKO) mice (*n* = 6 mice for vehicle group and *n* = 9 mice for DMXAA group) or in Ifnar1^fx/fx^ (WT) mice (*n* = 5 mice for vehicle group and *n* = 7 mice for DMXAA group), ^#^*P* = 0.0027 (*Ifnar1*^fx/fx^ + vehicle vs. *Ifnar1*^fx/fx^; Nav1.8-Cre + vehicle); *P* = 0.0011 (*Ifnar1*^fx/fx^ + vehicle vs. *Ifnar1*^fx/fx^; Nav1.8-Cre + DMXAA); *P* = 0.0020 (*Ifnar1*^fx/fx^ + DMXAA vs. *Ifnar1*^fx/fx^; Nav1.8-Cre + vehicle); *P* = 0.0006 (*Ifnar1*^fx/fx^ + DMXAA vs. *Ifnar1*^fx/fx^; Nav1.8-Cre + DMXAA); ***P < 0.001. **h**–**i** Measurement of mechanical allodynia by von Frey testing after DMXAA and ADU-S100 treatment (at d3 and d7, i.p.) in STING^fx/fx^; Na_v_1.8-Cre (STING-cKO) mice or STING^fx/fx^ (WT) mice. **h** Schematic of experimental design. **i** Paw withdrawal frequency. *n* = 6 mice for STING^fx/fx^; Na_v_1.8-Cre (Vehicle) group and *n* = 7 mice for the rest groups, ***P < 0.001. Notably, DMXAA and ADU-S100 attenuated mechanical allodynia at later time points (d10, d14) in STING-cKO mice. All data indicate the mean ± SEM, repeated-measures two-way ANOVA with Bonferroni’s *post-hoc* test (**a**–**i**). Source data are provided as a Source Data file.

Repeated administration of STING agonists may suppress cancer-induced bone pain by reducing tumor burden, reducing bone destruction, by a neuronal mechanism involving direct suppression of nociceptor activity, or a combination of all three of these mechanisms. Our electrophysiological data indicate that STING agonists can suppress bone cancer-induced pain via a direct neuronal mechanism which is independent to effects on tumor growth or bone destruction. To test whether acute administration of STING agonists can suppress bone cancer-induced pain, we performed behavioral testing in mice on d11 after tumor inoculation 4 h after single i.p. injection of vehicle, DMXAA, or ADU-S100. Notably, both STING agonists induced a substantial reduction in mechanical allodynia, cold allodynia, and spontaneous pain (Fig. 5a–c and Supplementary Fig. 5a, b). Since the natural activators of STING are intracellular double-stranded DNA (dsDNA), via a cGAS-dependent pathway, and via a cGAS-independent pathway involving bacterially-derived cyclic dinucleotides such as 3′3′-cGAMP^41^, we also assessed whether dsDNA and 3′3′-cGAMP could produce antinociception in the bone cancer model. Interestingly, i.p. administration of dsDNA (30 µg, complexed with LyoVec to facilitate cellular penetration) or cGAMP (20 mg/kg) could attenuate cold allodynia or/and mechanical allodynia 4 h after injection on d11 post LLC implantation (Supplementary Fig. 5c–f). Given that bone cancer pain is frequently treated by opioids such as morphine, we tested the analgesic potency of DMXAA relative to morphine (10 mg/kg). Notably, while both morphine and DMXAA could rapidly attenuate mechanical and cold allodynia with similar efficacy 1 h after administration, the effects of DMXAA were much longer lasting (Fig. 5b, c). In addition, we tested whether the acute antinociceptive effects of DMXAA were STING- and Ifnar1-dependent by injecting DMXAA (20 mg/kg, i.p.) into WT, STING^*gt/gt*^ mice and *Ifnar1*^*−/−*^ mice, measuring mechanical and cold allodynia 4 h after injection. We found DMXAA could reduce mechanical allodynia and cold allodynia in WT mice but not in STING^*gt/gt*^ mice or *Ifnar1*^*−/−*^ mice (Fig. 5d, e). To further determine whether neuronal IFN-I signaling is responsible for the acute antinociceptive effects of STING agonists, we established the bone cancer pain model using mice lacking *Ifnar1* selectively in sensory neurons (Ifnar1^fx/fx^; Na_v_1.8-Cre; Ifnar1-cKO), or their wildtype littermates. Importantly, we found the antinociceptive effects conferred by a single administration of DMXAA (20 mg/kg, i.p.) were present in WT, but not Ifnar1-cKO littermates (Fig. 5f–g). Given the immediacy of these effects, and taken in conjunction with our electrophysiological data, we conclude that STING agonists exert antinociceptive effects via direct actions on nociceptors in an Ifnar1-dependent mechanism.

To formally test the contribution of STING signaling in peripheral sensory neurons, we utilized mice lacking STING selectively in sensory neurons (STING^*fx/fx*^; Na_v_1.8-Cre; STING-cKO) or their wildtype littermates. Similar to STING^*gt/gt*^ mice, STING-cKO mice exhibit mechanical hypersensitivity at baseline relative to STING-WT littermate controls, which we previously reported^23^. Following innoculation with LLC cells via intrafemoral injection, STING-WT and STING-cKO mice were administered vehicle, DMXAA, or ADU-S100 at d3 and d7. Notably, behavioral testing was performed prior to DMXAA administration at d7 and again 4 h after administration, allowing us to assess the acute effects of STING agonist administration (Fig. 5h). Notably, while DMXAA and ADU-S100 both significantly attenuated mechanical allodynia 4 h after administration in STING-WT mice, this effect was abolished in STING-cKO mice, suggesting the acute antinociceptive effects rely on STING signaling in peripheral sensory neurons. Interestingly, however, the reduction in mechanical allodynia at later time points (d10, d14) was observed in both STING-WT and STING-cKO mice (Fig. 5i). These data demonstrate that the long-term protective effects of STING agonists are dependent on their feature of antitumor immunity.

### STING agonists suppress local bone cancer tumor burden and further metastasis

**Fig. 6:**
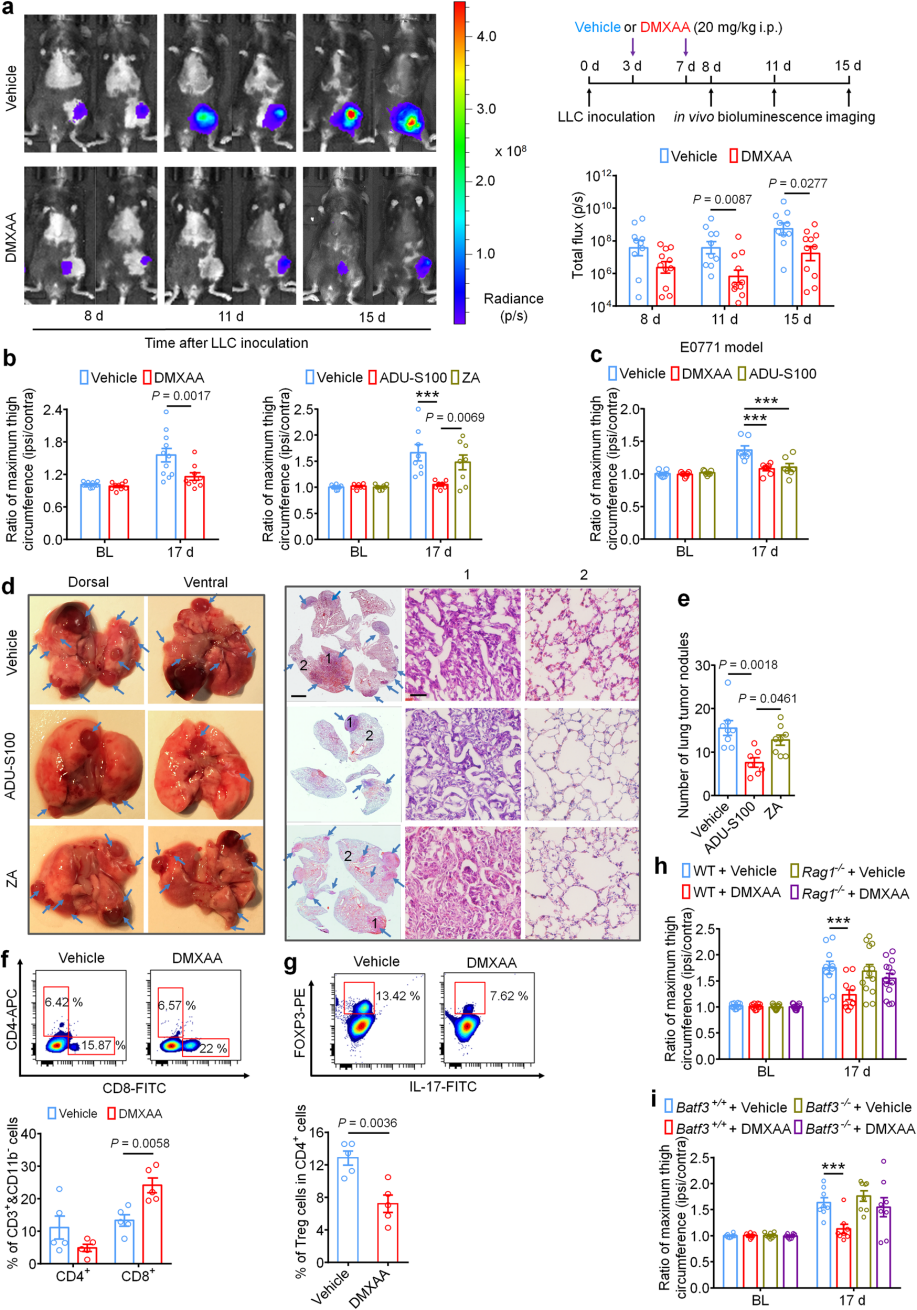
Systemic STING agonists reduce local tumor burden in the bone cancer tumor microenvironment. **a** In vivo bioluminescence of flux emitted by LL/2-Luc2 carcinoma (LLC) cells in tumor bearing femur after vehicle or DMXAA treatment (2 × 20 mg/kg, i.p.) measured at d8, d11, and d15 post tumor inoculation (*n* = 10 vehicle-treated mice, *n* = 11 DMXAA-treated mice). Images (left) were obtained at 15 min after i.p. injection of D-luciferin (30 mg/kg). Right, experimental scheme and quantification of **a**. **b** Ratio of maximum thigh circumference reflecting local tumor burden in mice with each indicated treatment on d17 after LLC implantation. Left, vehicle or DMXAA treatment (2 × 20 mg/kg, i.p.; *n* = 11 vehicle-treated mice and *n* = 9 DMXAA-treated mice). Right, vehicle, ADU-S100 (2 × 20 mg/kg, i.p.) or ZA (zoledronic acid; 2 × 100 µg/kg, i.p.) treatment (*n* = 8 vehicle-treated mice and *n* = 7 ADU-S100-treated mice, and *n* = 8 ZA-treated mice), ***P < 0.001. **c** Ratio of maximum thigh circumference in mice administered with vehicle, DMXAA or ADU-S100 (2 × 100 µg/kg, i.p.) on d17 after implantation of E0771 breast cancer cells (*n* = 7 mice/group), ***P < 0.001. **d** Images of lung tumor nodules in mice with each indicated treatment on d17 after LLC inoculation. Left, representative dorsal and ventral murine lung image, with arrows showing metastatic tumor nodules. Right, H&E staining for sections from lung samples in Left. Arrows indicates the areas with tumor cells, scale bar, 2 mm. 1 and 2 are enlarged images showing tumor tissue and peritumoral areas, respectively. Scale bar, 50 µm. Note that tumor cells have large and irregular nuclei with loss of the normal alveolar structure. **e** Quantification of panel d (*n* = 8 vehicle-treated mice, *n* = 7 ADU-S100-treated mice, and *n* = 8 ZA-treated mice). **f**-**g** FACS analysis of CD4^+^ and CD8^+^ T cells (**f**) or Treg cells (**g**) within the bone marrow tumor microenvironment in mice treated with vehicle or DMXAA (2 × 20 mg/kg, i.p.) on d8 post-LLC inoculation (*n* = 5 mice/group). The gating strategy for this figure is provided in Supplementary Fig. 7. **h** Local tumor burden as determined by the ratio of maximum thigh circumference after vehicle or DMXAA treatment in WT (*n* = 10 mice for vehicle or DMXAA group) and *Rag1*^*−/−*^ mice (*n* = 13 mice for vehicle or DMXAA group) on d17 after LLC implantation, ***P < 0.001. **i** Local tumor burden as determined by the ratio of maximum thigh circumference in *Batf3*^*+/+*^ and *Batf3*^*−/−*^ mice with indicated treatment measured on d17 after LLC implantation (*n* = 8 mice/group), ***P < 0.001. Data indicate the mean ± SEM, repeated-measures two-way ANOVA with Bonferroni’s *post hoc* test (**a**, **b**, **c**, **h**, **i**); one-way ANOVA with Bonferroni’s *post hoc* test (**e**); two-tailed Student’s *t*-test (**f**, **g**). Source data are provided as a Source Data file.

Intratumor injection of STING agonists have been reported to reduce tumor growth by promoting T cell-mediated antitumor immunity in several preclinical animal studies^12,17,20,42^. It is unknown, however, whether systemic administration of STING agonists can attenuate tumor progression in the bone marrow, which is generally regarded as an overwhelmingly immunosuppressive tumor microenvironment^24^. To answer this question, luciferase-labeled LLC cells (LL/2-Luc2 cell line) were used to establish the metastatic bone cancer model via intrafemoral inoculation, thereby enabling measurement of local tumor burden by in vivo bioluminescent imaging. Mice were treated with vehicle or DMXAA (20 mg/kg, i.p. at d3 and d7), followed by in vivo bioluminescence imaging at d8, d11, and d15. Notably, mice treated with DMXAA exhibited lower local tumor burden at d11 and d15, as measured by total flux of LL/2-Luc2 cells in tumor-bearing mice (Fig. 6a). By d17, tumor growth beyond the normal anatomic boundaries of the distal femur could be visually observed, leading to an increase in the circumference of the tumor-inoculated (ipsilateral) thigh compared to the contralateral side. To quantify this, we measured the ratio of the maximum thigh circumference (ipsilateral/contralateral), which accurately reflects local tumor volume^43^. Notably, we found that DMXAA and ADU-S100 treatment, but not ZA treatment, could reduce the ratio of maximum thigh circumference compared to the vehicle-treated group on d17 in both the LLC and E0771-induced bone cancer models (Fig. 6b, c). To test whether these effects were dependent on host-intrinsic STING and Ifnar1, we measured local tumor burden using thigh circumference at d17 in STING^*gt/gt*^ and *Ifnar1*^*−/−*^ mice and found that this protective effect was abolished in mice lacking either STING or *Ifnar1* (Supplementary Fig. 6a–c). Thus, systemic administration of STING agonists reduces local bone cancer tumor burden in a STING- and Ifnar1-dependent manner. However, the anti-tumor effects of DMXAA and ADU-S100 remained in STING-cKO mice with STING knockout in nociceptors (Supplementary Fig. S6d). This result suggests that the long-term protective effects of STING agonists depend on their antitumor immunity.

LLC is a murine lung adenocarcinoma cell line which has affinity to metastasize from the original injection site to pulmonary lobes and form visible tumor nodules^44^, enabling use of this phenomenon as a measure of metastasis in our model. To test whether systemic administration of ADU-S100 (20 mg/kg i.p.) or ZA (100 µg/kg i.p.) at d3 and d7 could reduce lung metastasis, we analyzed lungs from vehicle-, ADU-S100-, or ZA-treated mice at d17 after intrafemoral LLC inoculation. We found that mice receiving ADU-S100 exhibited fewer lung tumor nodules compared to mice treated with vehicle or ZA (Fig. 6d–e). Thus, systemic STING activation with ADU-S100 can inhibit both local tumor burden as well as further tumor metastasis.

Mechanistically, the antitumor effects of STING pathway activation are chiefly attributed to antigen presenting cell (APC)-mediated activation of CD8^+^ T cells^45^. To test whether systemic STING activation can promote CD8^+^ T-cell infiltration into the immunosuppressive tumor microenvironment of the bone marrow in our bone cancer model, mice were administered DMXAA (20 mg/kg i.p.) at d3 and d7 and bone marrow was collected from tumor-bearing femora 24 h after the second DMXAA injection for analysis of tumor-infiltrating lymphocytes (TILs) by flow cytometry (gating strategy provided in Supplementary Fig. S7). Importantly, we found that DMXAA treatment significantly increased the proportion of (CD11b^−^ CD3^+^) CD8^+^ T cells without significantly changing the proportion of (CD11b^−^ CD3^+^) CD4^+^ T cells in the bone marrow tumor microenvironment (Fig. 6f). We further analyzed the proportion of immunosuppressive (CD3^+^ CD4^+^) Foxp3^+^, IL-17^−^ regulatory T (Treg)cells and found that DMXAA treatment decreased the proportion of Treg cells in the bone marrow (Fig. 6g). To test whether STING agonist-induced reduction in tumor burden is due to T cell-mediated antitumor immunity, we introduced LLC cells into the intrafemoral cavity of WT or *Rag1*^*−/−*^ mice lacking mature B and T cells, followed by vehicle or DMXAA treatment (20 mg/kg i.p. at d3 and d7 post-inoculation) and measurement of maximum thigh circumference at d17 as in Fig. 6b. We found that DMXAA effectively reduced the ratio of maximum thigh circumference only in WT mice but not in *Rag1*^*−/−*^ mice (Fig. 6h).

Next, given that conventional type 1 dendritic cells (cDC1) have been demonstrated to be critical for cross-priming adaptive T cell responses against tumors through STING-mediated IFN-I induction, we also utilized cDC1-deficient *Batf3*^*−/−*^ mice to test whether the acute and/or long-term protective effects would depend on adaptive antitumor immunity. As expected, upon femoral inoculation of *Batf3*^*+/+*^ or *Batf3*^*−/−*^ mice with LLC cells, only tumor-bearing *Batf3*^*+/+*^ mice but not *Batf3*^*−/−*^ mice exhibited a decrease in local tumor burden at d17 following DMXAA treatment (Fig. 6i). Thus, we conclude that systemic activation of host-intrinsic STING-mediated IFN-I signaling facilitates antitumor immunity by promoting TIL entry into the normally immunosuppressive bone marrow TME.

To further observe the protective effect of STING agonist on bone cancer pain, we used a more physiological model of bone metastasis by administering LLC cells (200,000 in 100 μl) via intra-caudal artery injection (Supplementary Fig. 8a), which was recently reported to deliver cancer cells to the hind limbs and caudal vertebrae with high efficacy and cause bone destruction^46^. We found that repeated DMXAA treatment on day 3, 7, and 11 (Supplementary Fig. 8b) was also sufficient to attenuate bone destruction localized to the caudal vertebrae and reduced the ratio of vertebral fracture at d21 after LLC injection (Supplementary Fig. 8c, d). This verifies that STING agonist could protect cancer induced bone destruction. In the intra-caudal artery LLC injection model, which is a typical tumor metastasis model, we also found that DMXAA can reduce the number of lung tumor modules (Supplementary Fig. 8e). Furthermore, we found that acute treatment with DMXAA at d11 after tumor inoculation could potently suppress bone cancer-induced mechanical allodynia (Supplementary Fig. 8f).

### STING agonists inhibit cancer-induced osteoclast differentiation via IFN-I signaling

**Fig. 7:**
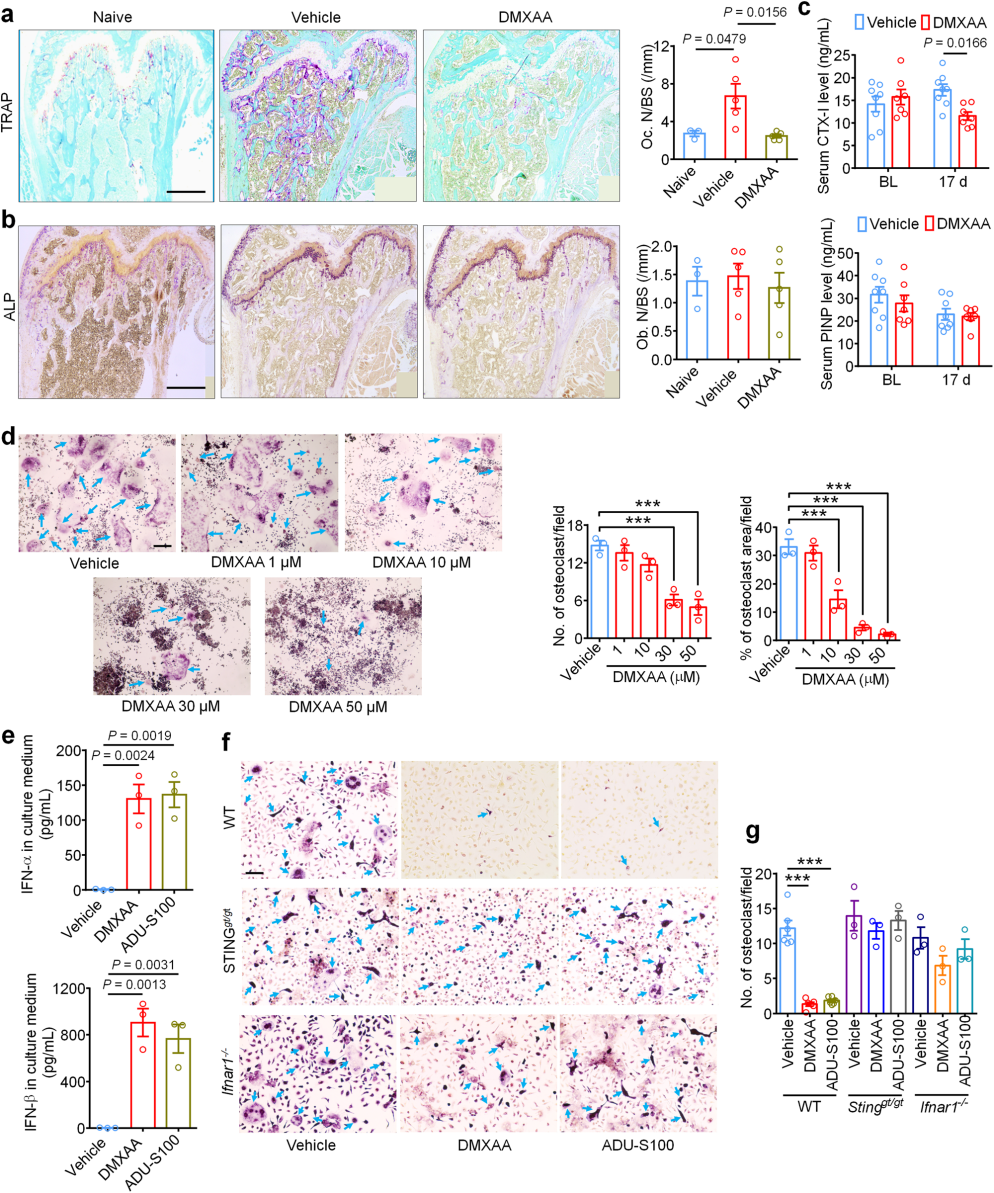
STING agonists attenuate cancer-induced osteoclastogenesis. **a** Representative images (left) and quantification (right) of TRAP staining to reveal osteoclast numbers in the tumor-bearing distal femora from mice treated with vehicle or DMXAA (2 × 20 mg/kg, i.p.) measured on d11 after LLC inoculation (*n* = 3 mice for naïve group and *n* = 5 mice for vehicle or DMXAA group). Scale bar, 500 µm. **b** Images (left) and quantification (right) of ALP staining to reveal osteoblasts in the tumor-bearing distal femora at d11 from mice with the indicated treatments (*n* = 3 mice for naïve group and *n* = 5 mice for vehicle or DMXAA group). Scale bar, 500 µM. **c** Measurement of serum CTX-I and PINP levels by ELISA at BL or d17 in vehicle or DMXAA (2 × 20 mg/kg, i.p.) treated mice (*n* = 8 vehicle-treated mice and *n* = 7 DMXAA-treated mice). **d** TRAP staining revealing osteoclast numbers after differentiation from RAW264.7 cells stimulated with 35 ng/ml RANKL, in the presence of increasing concentrations of DMXAA. Arrows indicate TRAP^+^ multinucleated osteoclasts. Left, representative TRAP-stained images. Right, quantification (*n* = 3 biologically independent experimental replicates), ***P < 0.001. Scale bar, 200 µm. **e** ELISA quantification of IFN-α and IFN-β levels in the culture medium of BMDM cells 24 h after DMXAA (30 µM) or ADU-S100 (30 µM) co-incubation. RANKL: 35 ng/ml, MCSF: 20 ng/ml (*n* = 3 biologically independent experimental replicates). **f**, **g** TRAP staining for osteoclasts differentiated from BMDM cells from WT mice, STING^*gt/gt*^ mice or *Ifnar1*^*−/−*^ mice, each treated with vehicle, DMXAA (30 µM) or ADU-S100 (30 µM). RANKL: 35 ng/ml, MCSF: 20 ng/ml. **f** Representative images of TRAP staining. Arrows indicate TRAP^+^ multinucleated osteoclasts. Scale bar, 100 µm. **g** Quantification for (**f**), ***P < 0.001. Sample sizes for **f**-**g** refer to independent cultures taken from individual mice and are as follows: WT/vehicle: *n* = 6, WT/DMXAA: *n* = 6, WT/vehicle: *n* = 6, STING^*gt/gt*^/vehicle: *n* = 3, STING^*gt/gt*^/DMXAA: *n* = 3, STING^*gt/gt*^/ADU-S100: *n* = 3, *Ifnar1*^*−/−*^/vehicle: *n* = 3, *Ifnar1*^*−/−*^/DMXAA: *n* = 3, *Ifnar1*^*−/−*^/ADU-S100: *n* = 3. Data indicate the mean ± SEM, one-way ANOVA with Bonferroni’s *post hoc* test (**a**, **b**, **d**, **e**, **g**); repeated-measures two-way ANOVA with Bonferroni’s *post-hoc* test (**c**). Source data are provided as a Source Data file.

IFN-α and IFN-β were previously reported to inhibit the differentiation of murine and human preosteoclasts into osteoclasts^26^. Given our data indicating that STING agonists can reduce bone destruction, we sought to determine whether the bone protective effects are mediated by direct effects on osteoclastogenesis. To this end, we measured osteoclast cell numbers in the distal tumor-bearing femora at d11 after inoculation in mice treated with vehicle or DMXAA (20 mg/kg i.p. at d3 and d7). Notably, DMXAA-treated mice exhibited far significantly fewer osteoclasts (Fig. 7a), but no changes were observed in bone-forming osteoblasts (Fig. 7b). To further evaluate the activity of osteoclasts and osteoblasts, we collected serum from tumor-bearing mice on BL and d17 after LLC inoculation and measured serum CTX-I and PINP levels, which are markers for bone resorption and bone formation, respectively^30,47^. DMXAA could effectively reduce CTX-I levels on d17 but had no effect on serum PINP levels (Fig. 7c). These data indicate that STING activation with DMXAA can suppress bone cancer-driven osteoclast formation and their bone catabolic activity.

Given that systemic STING agonist treatment reduces osteoclast numbers in vivo, we sought to determine whether STING pathway activation can promote osteoclast differentiation in vitro. Murine macrophage RAW 264.7 cells were treated with RANKL (35 ng/ml, for 6 days) to promote osteoclast differentiation^30^ in the presence of vehicle or an escalating dose of DMXAA or ADU-S100. Importantly, we found that both DMXAA and ADU-S100 dose dependently inhibited osteoclast differentiation (Fig. 7d, Supplementary Fig. 9a, b). Bone marrow cells from WT, STING^*gt/gt*^, or *Ifnar1*^*−/−*^ mice were harvested and differentiated into macrophages with 20 ng/ml M-CSF for 3 days. These bone marrow-derived macrophages (BMDM) were further induced into osteoclasts with 20 ng/ml M-CSF and 35 ng/ml RANKL for 7 days^30^. We collected the BMDM culture medium 24 h after incubation with DMXAA or ADU-S100 and found both agonists induced a drastic increase in IFN-α and IFN-β levels in the culture medium, although IFN-β induction was much greater (Fig. 7e). Furthermore, TRAP staining showed that DMXAA or ADU-S100 treatment (30 µM each) could significantly inhibit osteoclast formation from BMDM from WT mice but not from STING^*gt/gt*^ or *Ifnar1*^*−/−*^ mice (Fig. 7f, g). To provide further support that these effects were reliant on IFN-α/β signaling, anti-IFN-α (600 ng/ml) or anti-IFN-β (600 ng/ml) neutralizing antibodies were added to the induction medium of BMDM followed by analysis of osteoclast formation. We found that anti-IFN-β antibody could block the inhibition of osteoclast formation by DMXAA or ADU-S100 (Supplementary Fig. 9c–f). Taken together, these data indicate that activation of the STING/IFN-I signaling axis can inhibit osteoclastogenesis.

**Fig. 8:**
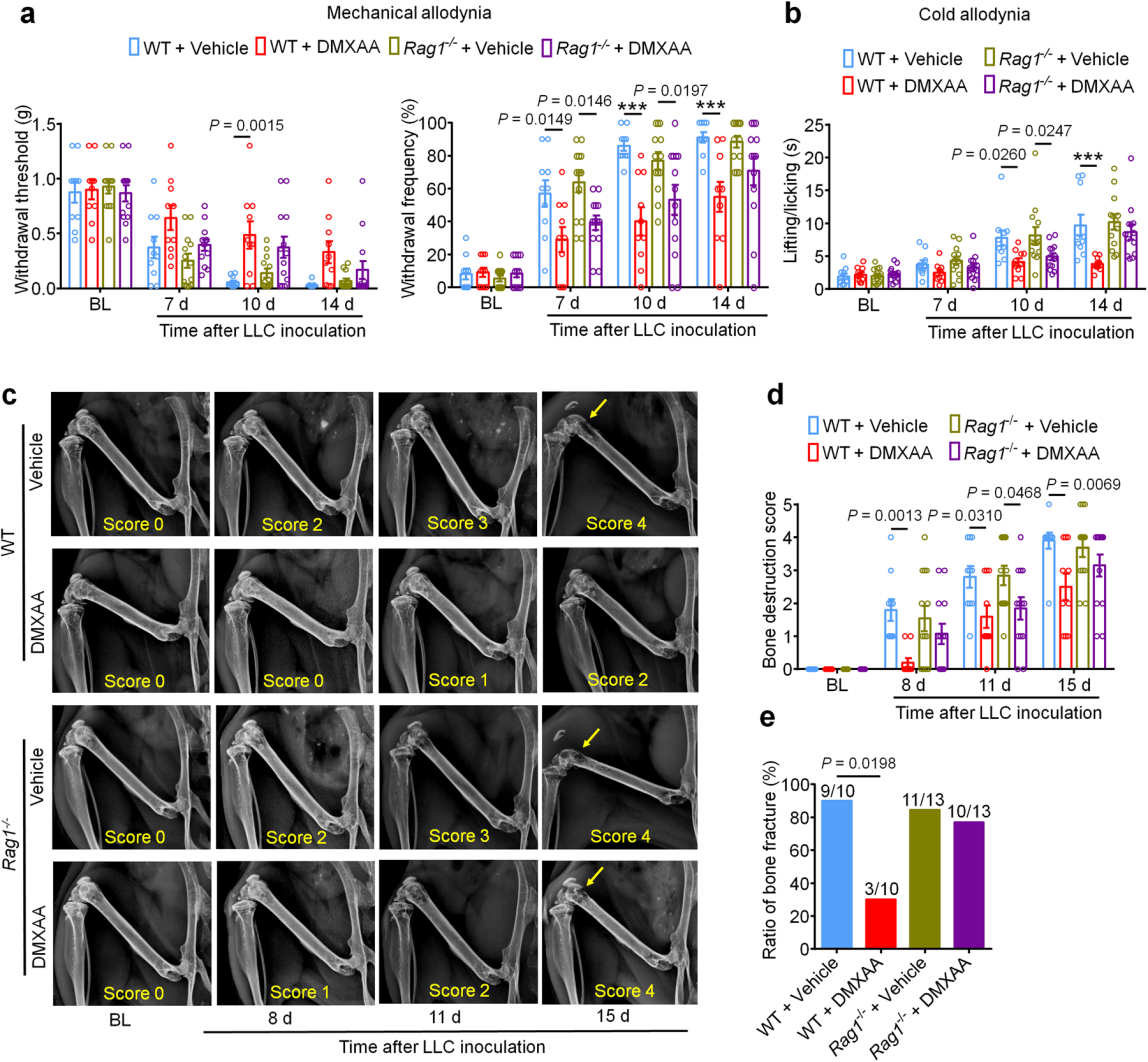
The analgesic and bone-protective effects of STING agonists largely retain in T cell-deficient *Rag1*^*−/−*^ mice. **a** Mechanical allodynia from von Frey test in WT or *Rag1*^*−/−*^ mice treated with vehicle or DMXAA (2 × 20 mg/kg, i.p.) on baseline (BL), day 7, 10 and 14 after tumor inoculation. Left, withdrawal threshold. Right, withdrawal frequency, ***P < 0.001. **b** Cold allodynia from acetone test in WT or *Rag1*^*−/−*^ mice with indicated therapy, ***P < 0.001. **c**, **d** Radiographical analysis of bone destruction in WT or *Rag1*^*−/−*^ mice administered vehicle or DMXAA, measured at BL, d8, d11 and d15 post LLC inoculation. **c** Representative X-ray images. Bone destruction score is labeled on the bottom of each photo and arrows indicate bone destruction scores of more than 3. **d** Quantification for (**c**). **e** Quantification of the proportion of mice with distal bone fractures, harvested and analyzed at d17 post-inoculation and with the indicated genotypes and treatment groups. Sample sizes for **a**–**e** were as follows: *n* = 10 vehicle-treated WT mice, *n* = 10 DMXAA-treated WT mice, *n* = 13 vehicle-treated *Rag1*^*−/−*^ mice, and *n* = 13 DMXAA-treated *Rag1*^*−/−*^ mice (pooled from two independent experiments). Data are Mean ± SEM, repeated-measures two-way ANOVA with Bonferroni’s *post hoc* test (**a**, **b**, **d**); two-sided Fisher’s exact test (**e**). Source data are provided as a Source Data file.

Our findings indicate that STING agonists produce antinociception, reduce tumor burden, and reduce bone destruction and osteoclastogenesis. One could argue that both the antinociceptive effects and the bone protective effects are secondary to T cell-mediated antitumor immunity. To test this possibility, we again introduced LLC cells into the intrafemoral cavity of WT or *Rag1*^*−/−*^ mice, followed by vehicle or DMXAA treatment (20 mg/kg i.p.) at d3 and d7 post-inoculation. Notably, bone cancer-induced mechanical and cold allodynia were reduced by DMXAA treatment in both WT and *Rag1*^*−/−*^ mice at early stages (d7 and d10), but not at later stages (d14; Fig. 8a, b). Likewise, DMXAA treatment led to an improvement in the bone destruction score in both WT and *Rag1*^*−/−*^ mice at d11, but not at d15 and at d17 for bone fracture (Fig. 8c–e). Thus, these data indicate that DMXAA suppresses pain and bone destruction in a T cell-independent mechanism at early stages, and thus, these effects are likely due to direct suppression of nociceptor excitability and osteoclastogenesis. As the local tumor burden increases at later stages, T cell-mediated antitumor immunity may become essential in controlling pain and bone destruction. To further verify these findings, we used *Batf3*^*+/+*^ and *Batf3*^*−/−*^ mice to establish bone cancer pain model. DMXAA treatment (20 mg/kg i.p., d3 and d7) could attenuate mechanical allodynia or cold allodynia in *Batf3*^*−/−*^ mice on d7 and d10 but not d14 after tumor inoculation (Supplementary Fig. 10a, b). DMXAA also reduced bone destruction on d8 and d11 but not d15 in *Batf3*^*−/−*^ mice (Supplementary Fig. 10c, d). These data provide an additional line of evidence that DMXAA suppresses pain and bone destruction in a T cell-independent mechanism at early stages.

### STING agonists inhibit fracture-induced bone pain in tumor-free mice

**Fig. 9:**
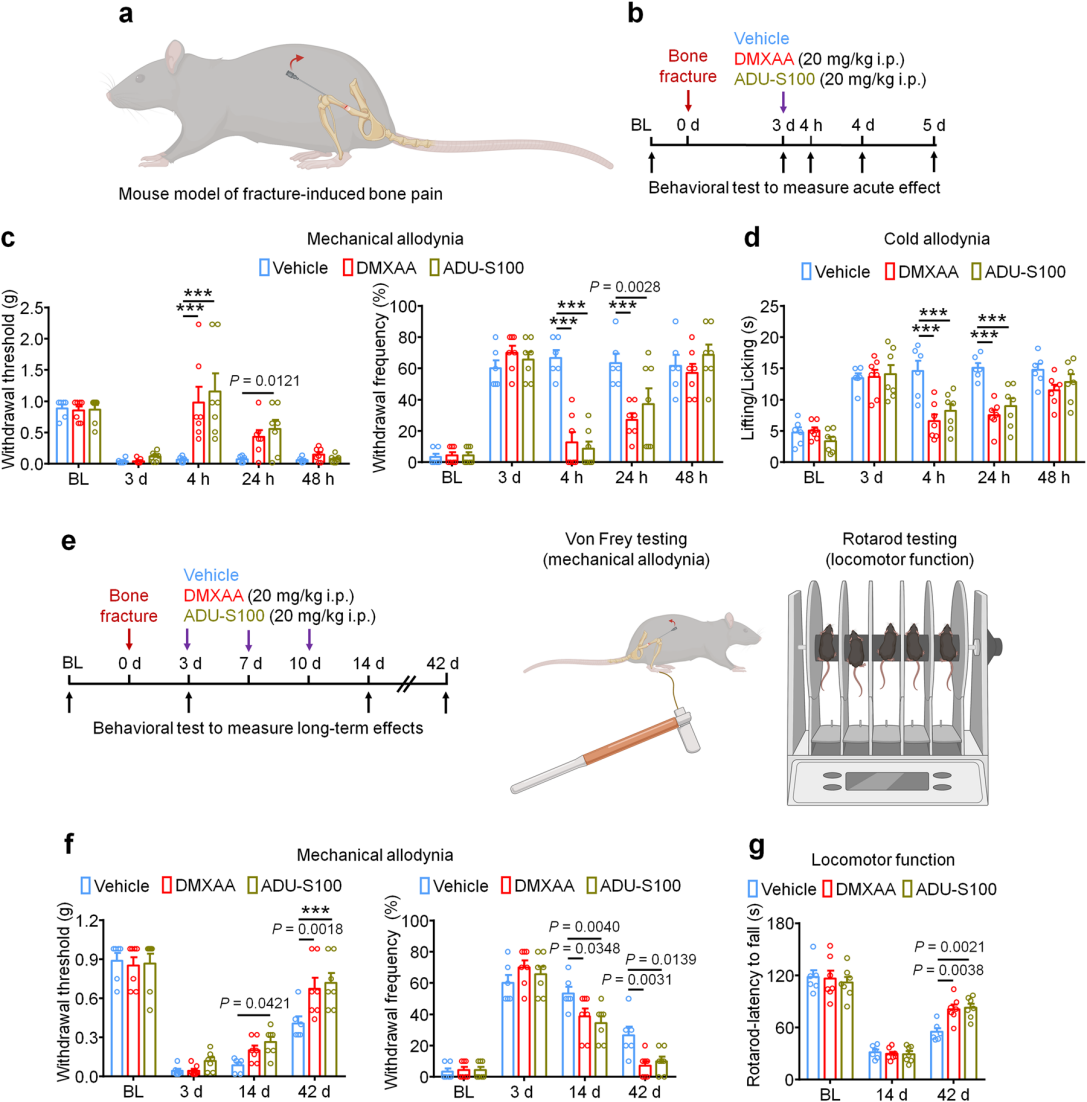
STING agonists reduce fracture-induced bone pain in tumor-free mice. **a** Schematic of mouse model of fracture-induced bone pain. **b** Experimental diagram indicating single treatment of vehicle, DMXAA, or ADU-S100 and behavioral testing. **c**, **d** Mechanical allodynia from von Frey test (**c**) and cold allodynia from acetone test (**d**) in mice treated with vehicle, DMXAA, or ADU-S100 (20 mg/kg i.p.) on d3 after bone fracture, ***P < 0.001. **e** Experimental diagram of repeated administration of vehicle, DMXAA, or ADU-S100 and behavioral testing for the measurement of long-term effects. **f** Von Frey test to determine mechanical allodynia, as assessed by withdrawal threshold (left) or withdrawal frequency (right) in mice treated with vehicle, DMXAA or ADU-S100 (3 × 20 mg/kg, i.p.) at d14 and d42 after bone fracture, ***P < 0.001. **g** Locomotor function of mice treated with vehicle, DMXAA or ADU-S100 (3 × 20 mg/kg, i.p.) at d14 and d42 after injury. Sample sizes for **c**, **d**, **f**, and **g** were as follows: *n* = 6 vehicle-treated mice, *n* = 7 DMXAA-treated mice, *n* = 7 ADU-S100-treated mice. Data are mean ± SEM, repeated-measures two-way ANOVA with Bonferroni’s *post hoc* test (**c**, **d**, **f**, **g**). Source data are provided as a Source Data file.

Finally, we used a bone fracture model which is a tumor-free model of bone pain to investigate the protective effects of STING agonists independent of their anti-tumor properties. The model was generated by controlled rotation of a rod which is inserted into the femoral cavity of the mouse femur (Fig. 9a). Given that this model generates intense pain nearly immediately following the injury, mice were treated with vehicle, DMXAA, or ADU-S100 3d (20 mg/kg i.p.) after bone fracture followed by behavioral testing to measure mechanical and cold allodynia (Fig. 9b). Interestingly, we observed that both DMXAA and ADU-S100 could potently suppress mechanical and cold allodynia for up to 24 h (Fig. 9c, d). To assess whether repeated administration of STING agonists in this model could facilitate pain resolution and functional recovery, mice were administered vehicle, DMXAA, or ADU-S100 (20 mg/kg i.p.) at d3, d7, and d10 after bone fracture, followed by measurement of mechanical allodynia and locomotor function at d14 and d42 (Fig. 9e). Notably, we found that DMXAA and ADU-S100 each conferred modest protective effects at d14 and d42 after injury (Fig. 9f) and robustly improved locomotor function as assessed by the rotarod test (Fig. 9g). Thus, in a cancer-independent model of bone pain, acute administration of STING agonists can attenuate pain transiently, while repeated administration of STING agonists can facilitate pain resolution and functional recovery following bone fracture.

**Fig. 10:**
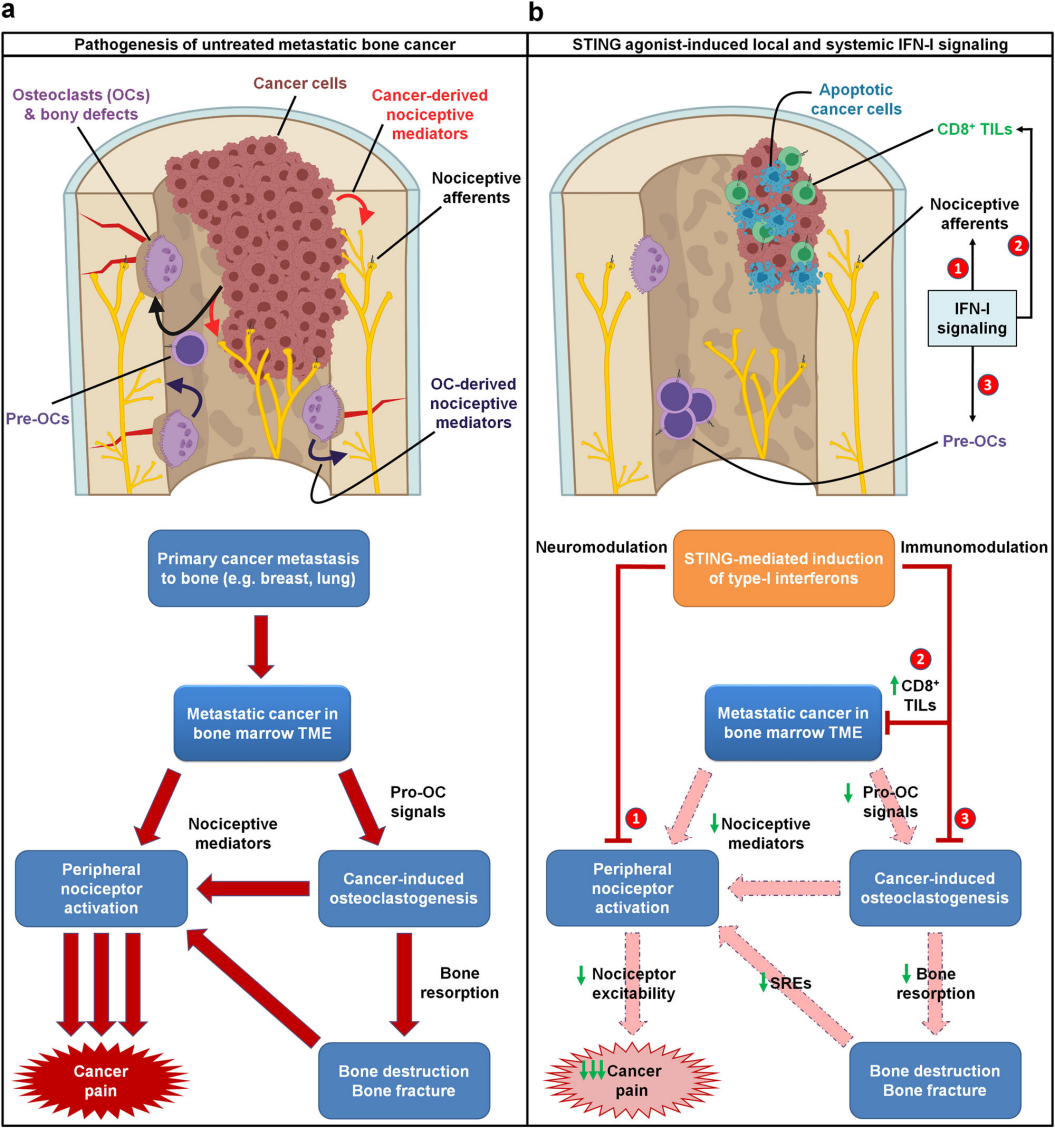
Schematic of mechanisms by which STING agonists directly and indirectly attenuate bone cancer-induced pain. **a** In untreated metastatic bone cancer, the pathophysiology driving cancer pain is multifaceted. Metastatic cancer cells present within the bone marrow tumor microenvironment (TME) produce pro-osteoclastogenic signals, driving cancer-induced osteoclastogenesis and osteoclast overactivation. Both cancer cells and osteoclasts produce nociceptive mediators, which directly activate peripheral nociceptive afferents present in the TME to produce pain through a direct mechanism. Additionally, cancer-induced osteoclast overactivation also leads to increased bone resorption, leading to increased bone destruction and bony fractures, which also produce pain through nociceptor activation. **b** STING agonists dramatically attenuate metastatic bone cancer-associated pain through multiple mechanisms, each mediated by host-intrinsic type-I interferon signaling. First, STING-mediated IFN-I signaling directly suppresses excitability of peripheral nociceptors (neuromodulation), leading to acute suppression of pain for the duration of the IFN-I response. In addition, STING-mediated IFN-I signaling promotes CD8^+^ T cell migration into the bone marrow TME, augmenting antitumor immunity and reducing tumor burden. In addition, IFN-I drives suppression of cancer-induced osteoclastogenesis. These two non-neuronal, immunomodulatory effects lead to sustained inhibition of pain by (1) reducing cancer cell- and osteoclast-derived pro-nociceptive mediators and (2) reducing osteoclast-mediated bone resorption, thereby attenuating subsequent bone destruction and bony fractures that frequently evoke pain and skeletal-related events (SRE). Thus, the immunomodulatory and neuromodulatory effects of STING agonists each individually suppress pain through actions on different cell types, and also synergistically suppress cancer pain through a convergence of shared downstream actions. Pre-OCs pre-osteoclasts, TILs tumor infiltrating lymphocytes.

Overall, we propose a mechanism by which STING agonists induce robust production of type-I interferons, which directly suppress nociceptor excitability and osteoclastogenesis while concurrently promoting T cell-mediated antitumor immunity. Thus, we posit that STING agonists can acutely suppress cancer pain through direct effects, while providing long term relief from bone cancer-induced pain by suppressing osteoclast-mediated bone destruction and relieving local tumor burden (Fig. 10).

## Discussion

Our findings elucidate a unique and synergistic advantage of STING agonism as a therapeutic strategy in treating metastatic bone cancer pain and its comorbidities, including tumor-induced bone destruction and functional impairment (e.g., immobility). To our knowledge, no other established or prospective immunotherapy agents have been demonstrated to possess combinatorial antitumor, antinociceptive, and bone anti-catabolic properties. Notably, while we demonstrate that STING-mediated IFN-I signaling exerts direct effects on pain via direct suppression of nociceptor excitability (neuromodulation), the potent analgesic effects of STING agonists in vivo are likely owed to a combinatorial suppression of tumor burden and bone destruction (immune modulation), and direct effects on nociceptive sensory neurons. In our intrafemoral inoculation model of bone cancer, tumor cells are directly introduced to the intrafemoral space (e.g. bone marrow), leading to a progressive pattern of cortical bone destruction as tumor cells cause increasing bone resorption and begin to invade tissues outside the bone cavity, frequently leading to fractures of the distal femur and severing of the tendon connecting the femur and patella. Given our data demonstrating that the STING/IFN-I signaling axis directly suppresses osteoclast differentiation, it is likely that STING agonists are particularly protective against bone cancer-induced bone destruction due to direct effects (e.g. Inhibition of osteoclastogenesis) and indirect effects (e.g. reduced tumor burden). These interrelated and synergistic protective actions of STING agonists (summarized in Fig. 10) would seem to be of particular advantage for the treatment of metastatic bone cancer, which offers unique challenges due to the ongoing presence of systemic disease in conjunction with cancer-associated SREs and severe pain which can themselves increase cancer mortality^48^. Regardless of the multitude of benefits that STING agonists seem to confer, it is important to consider the time point(s) at which intervention may be of therapeutic value. While our study focuses on relatively early administration of STING agonists (day 3 and day 7 post tumor-inoculation), the protective effects persist on bone cancer pain, bone destruction, and local tumor burden persist until relatively late time points. It is likely that early-stage intervention is more effective than late-stage intervention, and it is important to note that many patients with bone cancer are diagnosed at relatively late stages of the disease^49^. Thus, whether STING agonists can confer long-lasting protection when administered at middle to late stages of the disease is an interesting and important future direction.

Notably, STING agonists are sufficient to produce antinociception in naïve mice in a STING- and type-I interferon-dependent manner^23^. In a tumor-free bone pain model induced by tibial fracture, STING agonists potently reduced mechanical and cold hypersensitivity and further improved motor function (Fig. 9). These observations further indicate that STING controls pain at multiple levels through modulation of the activity of sensory neurons, osteoclasts, and cancer cells (Fig. 10). Interestingly, mice lacking STING and *Ifnar1* show significantly increased bone pain at baseline (mechanical allodynia), supporting a critical role of STING/IFN-I in the regulation of pain under steady-state conditions^23^. Given that STING is an integrator of many PRR signaling pathways, and is itself a sensor of DNA derived from commensal microflora, invading pathogens, and host cells, a key question remains: what are the signals that activate the STING/IFN-I signaling axis to influence physiological nociception? Interestingly, the commensal microbiota has been demonstrated to regulate basal levels of systemic IFN-I through toll-like receptor signaling, thereby priming protective immunity^50,51^. Whether a similar STING-mediated mechanism exists within sensory neurons to suppress physiological pain is an intriguing future direction. Similarly, within the bone marrow microenvironment, ample evidence suggests the gut microbiome exerts an important regulatory role on physiological bone homeostasis through osteoimmunomodulation, although whether the net effect is anabolic or catabolic remains debated^52,53^.

We note several limitations in this study. First, the increased pain sensitivity of STING and Ifnar1-deficient mice may complicate the interpretation of the antinociceptive effects of STING agonists. Nevertheless, STING agonists failed to raise pain thresholds in these knockout mice. Additionally, STING agonists may attenuate cancer pain and bone pain through actions on additional cell types not analyzed within the present study, such as peripheral immune cells or glial cells within the dorsal root ganglion or the spinal dorsal horn. Thus, an important future direction will be to investigate the specific immune cell and glial cell types that are involved in the analgesic actions of the STING/IFN-I pathway. Interestingly, microglia is the major cell type that expresses STING in the spinal cord dorsal horn^23^, and microglia are an important contributor to the pathogenesis of chronic pain, including cancer pain^54,55^. Third, our electrophysiological studies focused on how STING/IFN-I signaling altered the activity of small diameter nociceptors, primarily unmyelinated C-fibers, as these neurons are critical contributors to bone cancer pain^33^. However, it is also important to note that tumor cells sensitize and injure both unmyelinated and myelinated sensory fibers that innervate the marrow and mineralized bone^56^. Thus, it will also be of great interest to investigate if STING agonists also modulate A-fiber activities, as IFN-I receptor components are expressed by both C-fibers and A-fibers in mouse DRG^23,57^. Finally, given the growing emphasis on rigor and reproducibility in preclinical and clinical studies, it will be important for additional studies exploring the role of STING in cancer pain to be performed, both to validate the findings of the present study and explore the generalizability of STING agonists in additional models of cancer pain and other pain conditions.

The majority of clinical trials exploring the efficacy of STING agonists in human patients to date have focused on intratumoral administration, primarily in advanced stage solid tumors. While systemic administration may increase treatment-related adverse effects (TREs), increased toxicity may be acceptable in patients with metastatic disease and/or poor long-term prognosis, especially if coupled with attenuated cancer pain and improved function. Moreover, while the immune-mediated antitumor effects may be maximized by intratumoral administration of STING agonists, this route of administration may not be sufficient to yield the analgesic or bone anabolic effects we demonstrate here. Notably, in human patients STING agonists exhibit little efficacy as a monotherapy but have shown results when used in conjunction with anti-PD-1 immunotherapy regimens. In these studies, however, results are typically measured in terms of objective response rate (ORR) which only considers tumor size reduction. Notably, anti-PD-1 treatment in the LLC-induced murine bone cancer model alleviated cancer pain but did not change tumor burden^30^. Despite the controversy of STING agonists in controlling tumor growth in pre-clinical and clinical studies, our findings highlight the use of STING-based immunotherapies for treating cancer pain, especially bone cancer pain, due to multiple actions of the STING pathway in immune modulation, neuromodulation, and neuro-immune interactions (Fig. 10). Thus, clinical trials testing prospective STING immunotherapy agents should also consider measuring cancer-associated comorbidities, including parameters of functional impairment and pain, as improving the quality of life in cancer patients is equally important as extending the duration of survival.

## Methods

### Reagents

The following reagents were used in this study: DMXAA (Cayman Chemical, 14617), ADU-S100 (Chemietek, CT-ADUS100), 3’3’-cGAMP (Invivogen, tlr-nacga), poly(dA:dT)/LyoVec (Invivogen, tlrl-patc), Zoledronic acid (Cayman Chemical, 14984), mouse RANKL protein (R&D systems, 462-TEC), mouse M-CSF (R&D systems, 416-ML), anti-mouse IFN-α neutralizing antibody (PBL Assay Science, 32100-1), anti-mouse IFN-β neutralizing antibody (PBL Assay Science, 32400-1) and rabbit polyclonal IgG control (Biolegend, CTL-4112).

### Animals

Adult C57BL/6 J mice (males and females, 8–10 weeks) were used for all behavioral and biochemical studies. STING “goldenticket” knockout mice (Stock No: 017537), STING floxed/conditional knockout mice (STING^*fx/fx*^; strain #031670), Ifnar1 global knockout mice (Stock No: 028288), Rag1 knockout mice (Stock No: 002216), Ifnar1 conditional knockout mice (Ifnar1^fx/fx^; Stock No: 028256) and *Batf3*^*−/−*^ mice (Stock No: 013755) were purchased from the Jackson Laboratory and maintained on a C57BL/6 J background. Na_v_1.8-Cre mice, also maintained on a C57BL/6 J background, were a gift from Rohini Kuner (University of Heidelberg). Behavioral testing was performed comparing STING^*gt/gt*^ and *Ifnar1*^*−/−*^ mice with wildtype littermate controls. These mice were maintained at an AAALAC-approved Duke University facility with two to five mice housed in each cage maintained in a 12 h light-dark cycle with *ad libitum* access to food and water. Animals were randomly assigned to different experimental groups. Our previous studies using similar types of behavioral and biochemical analyses^30,58,59^ were used to determine sample size. Males and females were used in a sex and age-matched manner, if not otherwise specified in the figure legends. All mouse procedures were approved by the Institutional Animal Care & Use Committee (IACUC) of Duke University. Animal experiments were conducted in strict accordance with the National Institutes of Health Guide for the Care and Use of Laboratory Animals.

### Cell culture

Murine Lewis lung carcinoma cell line LL/2 (LLC1) (ATCC^®^ CRL-1642), luciferase expressing cell line LL/2-Luc2 (ATCC^®^ CRL-1642-LUC2™) and murine monocyte/macrophage cell line RAW 264.7 (ATCC^®^ TIB-71) were obtained from ATCC. The mouse E0771 breast cancer cell line (94A001) was obtained from CH3 BioSystems. Cells were cultured in high glucose (4.5 g/L) Dulbecco’s modified Eagle medium (Gibco, Thermo Fisher Scientific), supplemented with 10% fetal bovine serum (Gibco, Thermo Fisher Scientific) and 1% antibiotic-antimycotic solution (Sigma-Aldrich). These cells were then cultured in the presence of 5% CO_2_ at 37 °C. Blasticidin (2 µg/ml, Gibco, Thermo Fisher Scientific) was added into LL/2-Luc2 culture medium and removed 3 days before the inoculation of mice. No testing was performed for mycoplasma contamination.

### Bone cancer pain models through femoral or intra-caudal artery inoculation of cancer cells

The murine cell lines LLC1, LL/2-Luc2 or E0771 were lightly digested using 0.05% trypsin, followed by centrifugation to remove poorly digested cell clusters. Cells were then resuspended in PBS at a concentration of 1×10^8^ cells/ml. The inoculation was performed as previously described^30,60^. Briefly, mice were anesthetized with 4% isoflurane and the left leg was shaved and the skin disinfected with 10% povidone-iodine and 75% ethanol. A superficial incision (0.5–1 cm) was made near the knee joint, exposing the patellar ligament. A new 25-gauge needle was inserted at the site of the intercondylar notch of the left femur into the femoral cavity, which was then replaced with a 10 µL microinjection syringe containing a 2 µL suspension of tumor cells (2 × 10^5^) followed by 2 µL absorbable gelatin sponge solution to seal the injection site. The syringe contents were slowly injected into the femoral cavity over a 2-min interval. To prevent further leakage of tumor cells outside of the bone cavity, the outer injection site was sealed with silicone adhesive (Kwik-Sil, World Precision Instruments, US). Animals with surgery related movement dysfunction or with outside bone tumor injection were excluded from the study. To further observe the protective effect of STING agonist on bone destruction and bone cancer pain, we used a bone metastasis model by administering LLC1 cells (2 × 10^5^ in 100 μl) via intra-caudal artery injection^46^. DMXAA treatment (20 mg/kg, i.p) was given on day 3, 7, and 11 after the inoculation. Bone destruction to the femur and caudal vertebrae, tumor burden, lung metastasis, and mechanical pain were assessed. The animals with intra-caudal artery LLC1 inoculation were sacrificed on day 21.

### Bone fracture pain model

C57BL/6 J mice were anesthetized with 4% isoflurane and the left leg was shaved and the skin disinfected with 10% povidone-iodine and 70% ethanol. A superficial incision (0.5–1 cm) was made near the knee joint to expose the patellar ligament, followed by insertion of a 25-gauge metal rod at the site of the intercondylar notch of the left femur to enter into the femoral cavity approximately 5–6 mm. While stabilizing the leg, the rod was then rotated ~75 degrees until the bone fractured, which could be confirmed by tactile sensation, visual inspection, and a reflexive hindpaw flinch^61^.

### Drug treatment

DMXAA was dissolved in sterile PBS containing 0.75% NaHCO_3_ and ADU-S100 was dissolved in sterile PBS into 20 mg/ml, and they were further diluted 10-fold in sterile PBS prior to injection for in vivo experiments. All other reagents were dissolved in sterile saline or PBS. For experiments utilizing rabbit anti-IFN-α or -β neutralizing antibodies, a polyclonal rabbit IgG antibody served as the control. For single i.p. injection, drugs were injected on d11 after tumor inoculation. For twice i.p. injection, drugs were delivered on d3 and d7 or d10 after LLC implantation, as detailed in the results and/or figure legends. Morphine sulfate was obtained from WEST-WARD Pharmaceuticals and administered at a dose of 10 mg/kg via i.p. injection, which produces maximal analgesia without impairing motor function^60^.

### Behavior tests

All the behavioral tests were conducted in a blinded manner and performed during between the hours of 9:00–16:00. Animals were habituated in a light and humidity-controlled testing environment for at least 2 days prior to baseline testing. For Von Frey testing, mice were confined to individual 5 × 5 cm boxes placed on an elevated wire grid. A blinded experimenter stimulated their hindpaws using a series of von Frey filaments with logarithmically increasing stiffness (0.02–2.56 g, Stoelting). Each filament was applied perpendicularly to the central plantar surface. The 50% paw withdrawal threshold was determined using Dixon’s up-down method^62^. In addition, we also measured paw withdrawal frequency to repeated stimulation (10 times, with ~1–2 minutes between each stimulation) using a subthreshold 0.16 g von Frey filament, which is a more sensitive method to detect mechanical allodynia. To measure cold allodynia, mice were similarly isolated to individual boxes on an elevated mesh floor, and a drop (~20–30 µl) of acetone was applied to the plantar hindpaw. The duration of time that animal displayed a nociceptive response (lifting or licking the paw) over a 90 s period immediately after acetone application was recorded. To measure locomotor function, we performed open field testing in which mice were placed in the center of a 45 × 45 cm chamber and locomotor activity was recorded by an overhead webcam connected to a laptop computer, and animals’ movements were automatically tracked for 30 min using ANY-Maze. The total distance traveled and mean speed during the 30-min period were analyzed. We also measured locomotor function using rotarod testing, which reflects locomotor impairment as well as motor coordination. Mice were placed in the behavioral room for 30 min prior to testing, followed by trials using an accelerating protocol (4-45 RPM over 300 s). Each mouse was tested in 3 daily sessions, each of which consisted of 3 independent trials which were separated by at least 10-minute intervals. The data displayed at baseline represent the average fall latency on the 3rd day of testing.

### In vivo X-ray radiography

Osteolytic bone destruction was continuously evaluated by radiography using the MultiFocus by Faxitron system (Faxitron Bioptics LLC, Tucson, Arizona). Radiographs of tumor-bearing femora were rated for bone destruction on a 0–5 score scale based on previous study^30^: 0 for normal bone at baseline without tumor inoculation; 1 for one to three radiolucent lesions indicative of bone destruction compared to baseline; 2 for increased number of lesions (three to six lesions) and loss of medullary bone; 3 for loss of medullary bone and erosion of cortical bone; 4 for full-thickness unicortical bone loss; 5 for full-thickness bicortical bone loss and displaced skeletal fracture. All radiographic image quantifications were completed by an experimenter who was blinded to the experimental conditions.

### Microcomputed tomography

Microcomputed tomography (MicroCT) analyses were performed on femurs from tumor inoculated mice or naïve mice using a VivaCT 80 scanner with the 55-kVp source (Scanco, Southeastern, PA) as previously described^30,63,64^. Quantification of microCT data was calculated for distal femurs of mice treated with vehicle or DMXAA. Parameters quantified included bone volume/total volume (BV/TV), connectivity density (Conn.D), trabecular number (Tb.N), trabecular thickness (Tb.Th), and trabecular separation (Tb.Sp) within a region of 100 slides and 200 slides proximal to the distal growth plate.

### Bone histology, TRAP, and ALP staining on mouse femurs

Mice were deeply anesthetized and perfused intracardially with 4% paraformaldehyde (PFA) in 0.1 M phosphate buffered saline. The femora were removed and then post-fixed for 48 h in the same fixative at 4 °C. After demineralization in EDTA (10%) for 10 days, femur samples were dehydrated in an ascending gradient of ethanol (30–100%) followed by paraffin embedding. Serial sections for trabecular bone were obtained from the distal femur at a thickness of 5 µm followed by tartrate-resistant acid phosphatase (TRAP) staining or alkaline phosphatase (ALP) staining using TRAP kits (Fast Red TR/Naphthol AS-MX, Sigma, St. Louis, MO) and NBT/BCIP (Thermo Scientific), respectively^30^. Bone static histomorphometric analyses for osteoclast number (osteoclast number per trabecular bone surface covered by osteoclasts, Oc.S/BS) and osteoblast number (osteoblast number per trabecular bone surface, Ob.N/BS) were conducted using Image J (NIH) based on images taken by a Leica Q500MC microscope. Osteoclasts, osteoblasts and trabecular bone at the metaphysis of the femur (1500 µm proximal to the distal growth plate) were quantified, since bone destruction in this model mainly occurs in this area^36,65^. Three sections per animal were randomly chosen and used for quantification.

### Visual and immunohistochemical analysis of lung metastases

Mice were deeply anesthetized with isoflurane and perfused intracardially with PBS, followed by 4% PFA. After the perfusion, lungs were removed from mice and post-fixed in the same fixative overnight. The samples were then dehydrated with a 30% sucrose solution, embedded in O.C.T. (Tissue Tek), and cryosectioned to produce 8 μm thick sections. For Hematoxylin and Eosin (H & E) staining, lung sections were rehydrated and stained with 0.1% Hematoxylin and 0.5% Eosin in sequence. After dehydration and clearance with HistoClear (Electron Microscopy Sciences, Hatfield, PA), slides were mounted with a resinous mounting medium (Mercedes Medical, Sarasota, FL) and subsequently imaged using a Leica Q500MC microscope with a digital camera at different magnifications.

### Bone marrow collection

Mice were humanely euthanized and femora were carefully removed and placed on ice. The muscles surrounding the femur were gently removed as much as possible, followed by separation of the distal epiphysis from femoral shaft. The proximal end of the femur removed, followed by insertion of a 25-gauge needle into the distal end of the femur and injection of 1 ml cold PBS or α-MEM (Gibco, Thermo Fisher Scientific) by lightly pressing down on the plunger, allowing the bone marrow to be evacuated into a 1.5 ml centrifuge tube. Bone marrow was harvested through centrifugation at 800 × *g* for 5 min at 4 °C for subsequent detections or cell cultures^30^.

### ELISA

Mouse high-sensitivity IFN-α ELISA kit (42115-1) and IFN-β ELISA kit (42410-1) were purchased from PBL Assay Science. Mouse CTX-I ELISA kit (AC-06F1) and mouse PINP ELISA kit (AC-33F1) were purchased from Immunodiagnostic Systems. ELISA was performed using culture medium, serum, and bone marrow lysates. Serum was obtained from whole blood that was collected from a submandibular vein via facial vein puncture, coagulated for 30 min at room temperature, followed by centrifugation (2000 × *g* for 10 min, 4 °C) and collection of the supernatant (serum). Bone marrow was homogenized in a lysis buffer containing protease inhibitors at 4 °C for 30 min. ELISA was conducted in accordance with the manufacturer’s instructions. A standard curve was performed for each experiment.

### In vitro induction of osteoclastogenesis and TRAP staining

For in vitro drug treatment, the RAW 264.7 cells were incubated with 35 ng/ml RANKL for six days. Isolated bone marrow cells were cultured overnight in α-MEM media containing 10% fetal bovine serum and 1% antibiotic-antimycotic solution. The suspended cells were collected and incubated with 20 ng/ml MCSF for 3 days to obtain bone marrow-derived macrophage (BMDM). The attached cells were further activated by 35 ng/ml RANKL and 20 ng/ml MCSF. Drug treatment was performed in tandem with RANKL. Culture medium and co-cultured reagents were changed every 3 days. After 6 or 7 days of incubation, the cells were fixed by 4% PFA and stained with warm TRAP staining solution (TRAP kit, Sigma-Aldrich, SLBW4002) for 10–30 min at 37 °C^30^. TRAP-positive multinucleated cells that displayed three or more nuclei under a light microscope were considered osteoclasts, and the numbers of positive cells were counted in a blinded fashion with images of randomly selected visual fields (4–5 regions per well) using Image J software.

### Flow cytometry

For analysis of immune subsets present within the bone marrow tumor microenvironment, bone marrow was collected followed by removal of RBC cells using RBC lysis buffer (Sigma, R7757). Cells were subsequently washed with PBS and resuspended in 2% paraformaldehyde in PBS for 10 min. Fixed cells were washed several times with PBS, followed by incubation in blocking buffer (1% anti-mouse-CD16/CD32, 2.4 G2, 2% FBS, 5% NRS,1% triton x100 and 2% NMS in HBSS; BD Bioscience) for 1 h at room temperature. Cells were subsequently stained with IL-17-FITC (1:20, rat, Miltenyi Biotec, 130-102-262), CD-3 APC/cy7 (1:200, rat, Biolegend, 100221), CD-4 APC (1:200, rat, Biolegend, 100411), FoxP3-PE (1:20, human, Miltenyl Biotec, 130-111-678), CD8a-FITC (1:200, rat, Biolegend, 100705), and CD11b-PE(1:200, rat, Biolegend, 101207) in blocking buffer for 1 h at room temperature. After staining, cells were washed in PBS with EDTA. Flow cytometry events were acquired by a BD FACS Canto II flow cytometer using the BD FACS Diva 8 software (BD Bioscience). Data were analyzed using Cytobank software (https://www.cytobank.org/cytobank). The gating strategy is provided in Supplementary Fig. 7.

### In vivo bioluminescence imaging

RediJect D-Luciferin Ultra was purchased from PerkinElmer (770505). Prior to in vivo imaging, mice were shaved in the region of interest depicted in the figure. Bioluminescence images of LL/2-Luc2 bearing mice were captured with IVIS Lumina III system 15 min after intraperitoneal injection of D-Luciferin (30 mg/kg)^30^. The IVIS acquisition control panel was set to following conditions for imaging: Exposure time = auto, Binning = medium, F/Stop = 1, Emission Filter = open. The bioluminescence images were analyzed using Living Image software from PerkinElmer.

### Whole-cell patch clamp recordings in whole-mount DRGs ex vivo

Four-week-old male C57BL/6 mice were used to establish the bone cancer pain model by intrafemoral inoculation of LLC, leading to nociceptor hyperexcitability in the ipsilateral L3–L5 DRGs, which extend afferent nerve fibers to the tumor-bearing femur. Notably, young mice were used for these experiments due to technical limitations in performing electrophysiological recordings on older mice. 11d after tumor implantation, mice were euthanized followed by careful isolation of L3-L5 DRGs, which were placed in oxygenated artificial cerebrospinal fluid. DRGs were lightly digested for 20 minutes using an enzymatic mixture consisting of 0.32 ml collagenase A (1 mg/mL) and Trypsin (0.25%). Intact DRGs were then incubated in ACSF oxygenated with 95% O_2_ and 5% CO_2_, supplemented with vehicle (PBS) or 30 µM DMXAA in PBS for 2 hours at 37 °C. Following incubation, DRGs were transferred to a recording chamber, where neurons could be visualized using a 40x water-immersion objective on an Olympus BX51WI microscope. Patch pipettes were pulled from borosilicate capillaries (Chase Scientific Glass Inc.) and filled with a pipette solution containing (in mM): 126 potassium gluconate, 10 NaCl, 1 MgCl_2_, 10 EGTA, 2 Na-ATP, and 0.1 Mg-GTP, adjusted to pH 7.3 with KOH. The external solution was composed of (in mM): 140 NaCl, 5 KCl, 2 CaCl_2_, 1 MgCl_2_, 10 HEPES, 10 glucose, adjusted to pH 7.4 with NaOH. The resistance of pipettes was 4-5 MΩ. Series resistance was compensated for (>80%) and leak subtraction was performed. Data were low-pass filtered at 2 KHz and sampled at 10 KHz. Data were recorded and analyzed using the pClamp10 (Axon Instruments) software. For each mouse, only a single neuron was recorded from each DRG. Successful recordings were performed on DRG neurons taken from 4/4 naïve mice (8 neurons total; 2 neurons from animal 1, 2 neurons from animal 2, 1 neuron from animal 3, and 3 neurons from animal 4), 4/6 mice bearing tumors for the vehicle-treated mice (9 neurons total; 3 neurons from animal 1, 3 neurons from animal 2, 1 neuron from animal 3, and 2 neurons from animal 4), and 4/6 mice bearing tumors for the DMXAA-treated mice (10 neurons total; 2 neurons from animal 1, 3 neurons from animal 2, 2 neurons from animal 3, and 3 neurons from animal 4).

### Data analysis and statistics

The sample sizes for each experiment were based on our previous studies on such experiments^30,58^. Statistical analysis was performed with GraphPad Prism 6.0 (GraphPad Software). All the data in the figures are expressed as mean ± standard error (SEM). Biochemical and behavioral data were analyzed using a two-tailed *t*-test (two groups), One-Way ANOVA, or Two-Way ANOVA, followed by post-hoc Bonferroni test. Fisher’s exact test was utilized for the comparison of the bone fracture ratio. The criterion of *P* < 0.05 was defined as the threshold for statistical significance. In each figure, significance denotes *P* values as follows: ***P < 0.001. See more statistical details in Statistical details sheet of Source Data file.

### Study approval

The present studies in animals were reviewed and approved by the Duke University Institutional Animal Care and Use Committee (IACUC). All animal procedures were conducted in accordance with the NIH’s Guide for the Care and Use of Laboratory Animals (National Academies Press, 2011).

### Reporting summary

Further information on research design is available in the Nature Research Reporting Summary linked to this article.

## Supplementary information

Supplementary information

Reporting Summary

## Data Availability

All data supporting the findings of this study are available within the Article, Supplementary Information or Source Data file. Source data are provided with this paper.

## Source data

Source data
