# Supplementary material for: STING suppresses bone cancer pain via immune and neuronal modulation
Source: Nat Commun. 2021 Jul 27;12:4558. doi: 10.1038/s41467-021-24867-2 (PMC8316360; doi:10.1038/s41467-021-24867-2)
Supplement: Supplementary file 1 — Supplementary information [file 41467_2021_24867_MOESM1_ESM.pdf]

Supplementary information

Wang et al. *Nature Communications*. STING suppresses bone cancer pain via immune and neuronal modulation (2021).

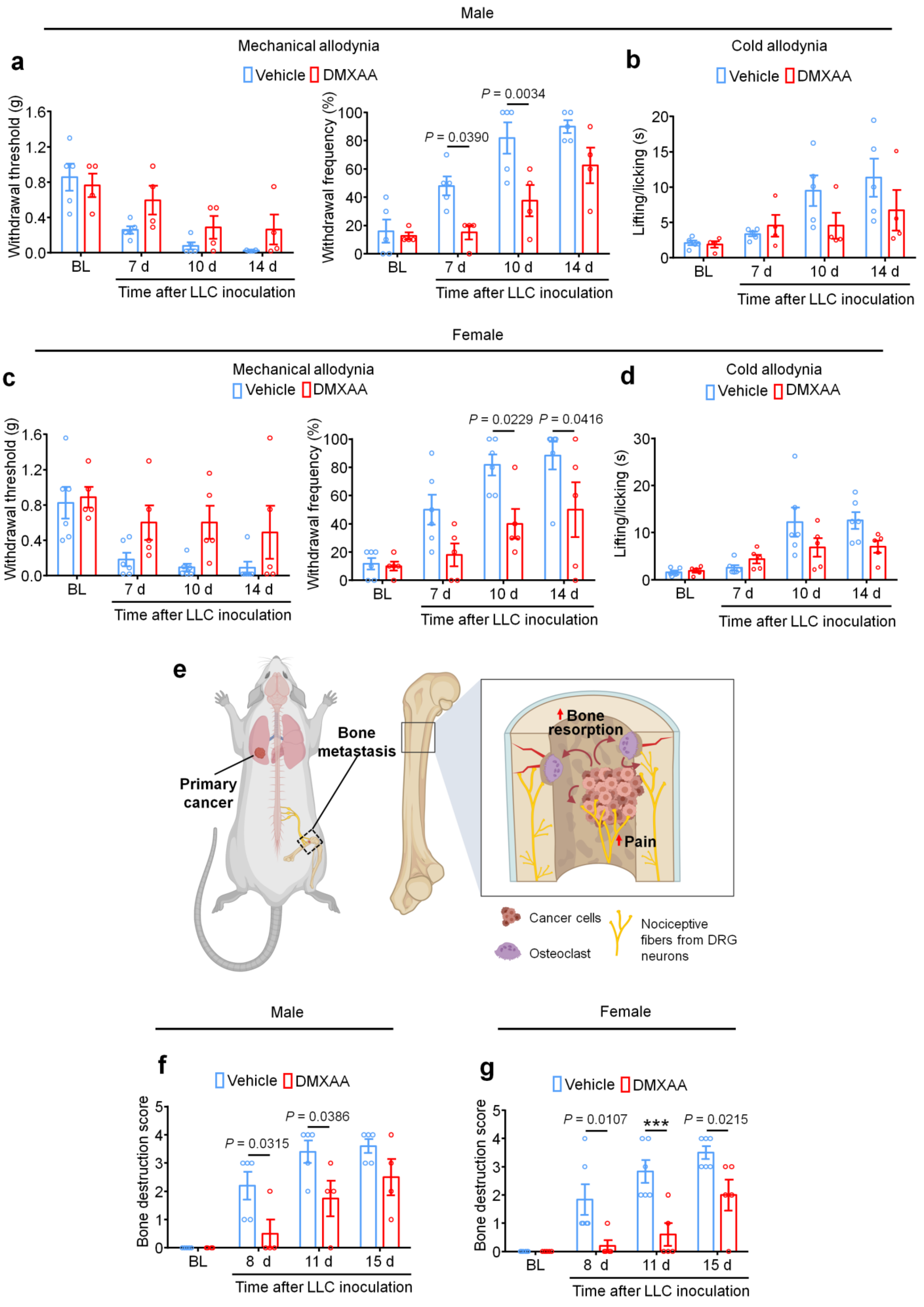

Male
a
Mechanical allodynia
Vehicle DMXAA
Withdrawal threshold (g)
1.6
1.2
0.8
0.4
0.0
BL
7 d
10 d
14 d
Time after LLC inoculation
Withdrawal frequency (%)
100
80
60
40
20
0
P = 0.0390
P = 0.0034
b
Cold allodynia
Vehicle DMXAA
Lifting/licking (s)
20
15
10
5
0
Female
c
Mechanical allodynia
Vehicle DMXAA
P = 0.0229
P = 0.0416
d
Cold allodynia
Vehicle DMXAA
30
e
Primary cancer
Bone metastasis
Bone resorption
Pain
Cancer cells
Osteoclast
Nociceptive fibers from DRG neurons
Male
Female
f
Vehicle DMXAA
Bone destruction score
P = 0.0315
P = 0.0386
8 d
11 d
15 d
g
P = 0.0107
***
P = 0.0215

Supplementary Fig. 1. STING agonists reduce bone cancer pain and bone destruction in both male and female mice.

a-d. Von Frey testing to determine mechanical allodynia (**a, c**) or acetone testing to determine cold allodynia (**b, d**) in male mice (**a, b**) and female mice (**c, d**), displayed separately. **e**. Schematic showing the mechanisms of bone cancer-induced pain, which originates from direct activation of local nociceptive afferents by mediators produced from cancer cells and osteoclasts as well as indirect mechanisms owed to osteoclast-induced bone resorption leading to subsequent bony fractures and breaks. **f-g**. Radiographic measurement of bone destruction following vehicle or DMXAA (2 x 20 mg/kg, i.p.) treatment at the indicated timepoints in male (**f**) or female mice (**g**), ***$P < 0.001$. Sample sizes are as follows: $n = 5$ vehicle-treated male mice, $n = 4$ DMXAA-treated male mice (panels **a, b, g**); $n = 6$ vehicle-treated female mice, and $n = 5$ DMXAA-treated female mice (panels **c, d, f**). Data displayed are the mean ± SEM, repeated-measures two-way ANOVA with Bonferroni's *post-hoc* test (**a, b, c, d, f, g**). Source data are provided as a Source Data file.

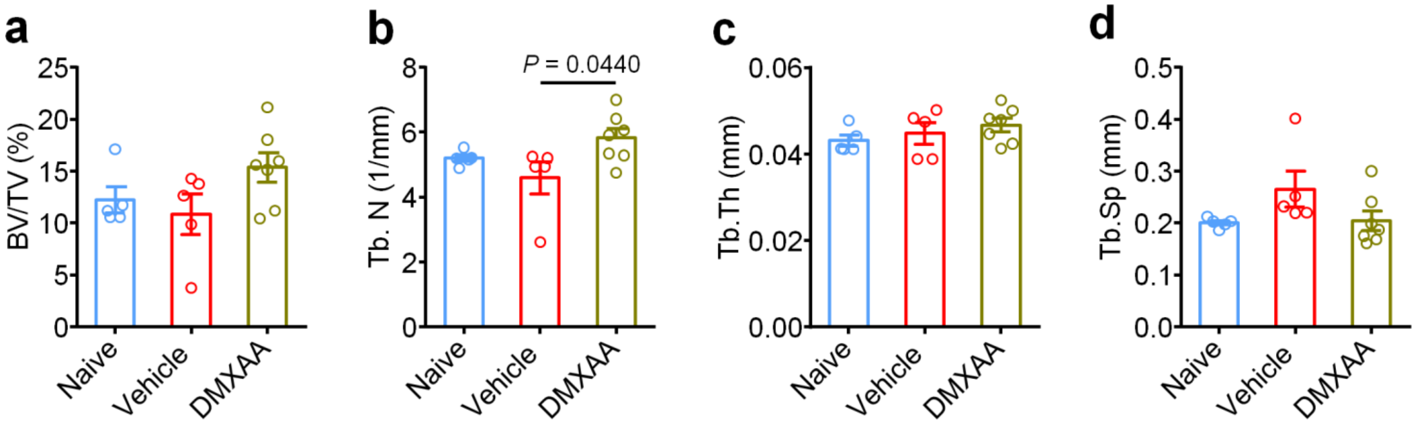

Supplementary Fig. 2. Comparison of microstructural parameters from trabecular bone between vehicle and DMXAA treated mice. **a-d**. Morphometric quantification of micro-CT images with analysis of BV/TV (**a**), trabecular number (Tb.N) (**b**), trabecular thickness (Tb.Th) (**c**) and trabecular separation (Tb.Sp) (**d**) in vehicle or DMXAA-treated mice (n = 5 naïve mice, n = 5 vehicle-treated mice, and n = 7 DMXAA-treated mice). Data are mean ± SEM, two-tailed Student's t-test. Source data are provided as a Source Data file.

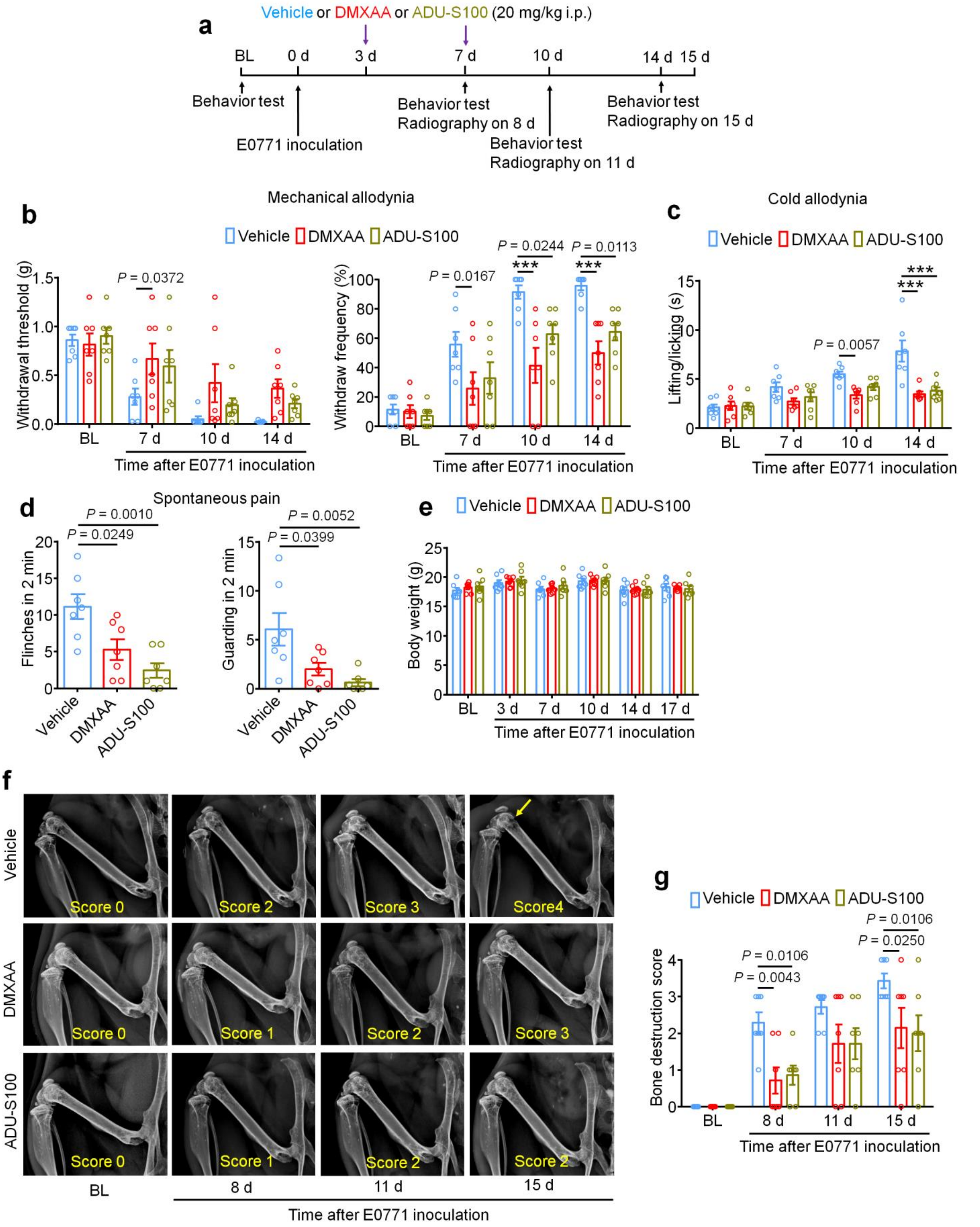

Supplementary Fig. 3. STING agonists confer protection in bone cancer induced by the breast cancer cell

line E0771.

a. Experimental diagram indicating vehicle, DMXAA, or ADU-S100 treatment, behavioral testing, and radiography. **b.** von Frey testing to determine withdrawal threshold (left) and frequency (right) from female mice treated with vehicle, DMXAA or ADU-S100 (*n* = 7 mice/group), ****P* < 0.001. **c.** Cold allodynia testing performed at BL, d7, d10 and d14 after E0771 inoculation (*n* = 7 mice/group), ****P* < 0.001. **d.** Spontaneous pain as quantified by number of flinches (left) or guarding (right) behaviors over a 2 minute interval at d14 after E0771 inoculation in mice with the indicated treatments (*n* = 7 mice/group). **e.** Measurement of body weight in mice with the indicated treatment (*n* = 7 mice/group). **f-g.** Representative X-ray images of tumor bearing femora (**f**) and quantification (**g**) of bone destruction score from radiography test on BL, d8, d11 and d15 post-E0771 implantation (*n* = 7 mice/group). Bone destruction score is indicated in each image and arrows show bone lesions with scores over 3. Data indicate the Mean ± SEM, repeated-measures two-way ANOVA with Bonferroni's *post hoc* test (**b, c, e, g**); one-way ANOVA with Bonferroni's *post-hoc* test (**d**). Source data are provided as a Source Data file.

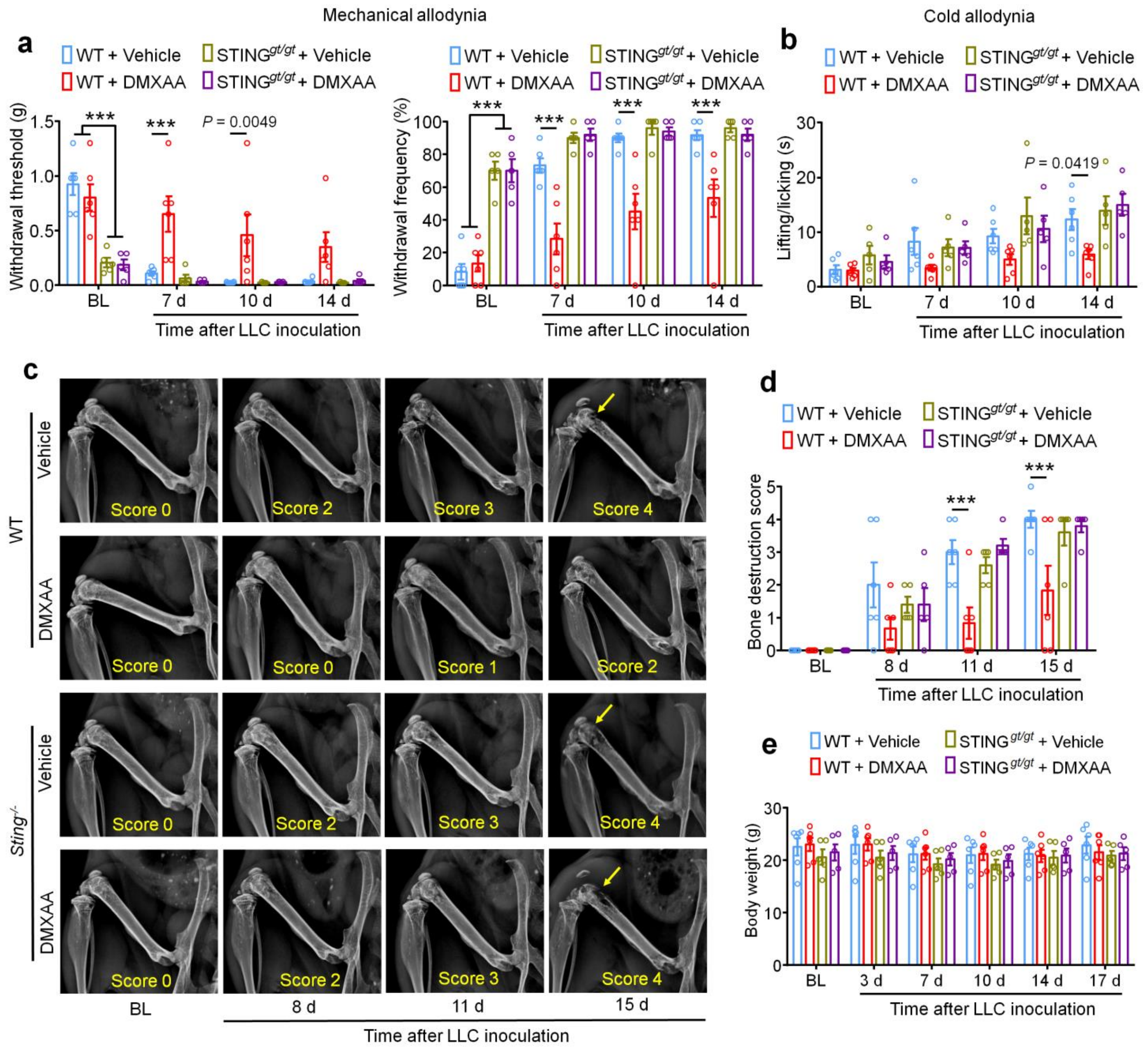

Supplementary Fig. 4. The protective effects of DMXAA treatment are STING dependent

a. Mechanical allodynia from von Frey tests in STING$^{+/+}$ (WT) and STING$^{gt/gt}$ mice treated with vehicle or DMXAA (2x20 mg/kg, i.p.). Left, withdraw threshold. Right, withdrawal frequency. ****P* < 0.001. **b.** Cold allodynia measured by acetone testing in STING$^{+/+}$ (WT) mice and STING$^{gt/gt}$ mice with the indicated treatments. **c-d.** Radiography to measure bone destruction score at the indicated timepoints after tumor inoculation in STING$^{+/+}$ (WT) and STING$^{gt/gt}$ mice with vehicle or DMXAA treatment. (**c**) Representative radiographs of tumor-bearing femora. Bone destruction score is indicated in each image and arrows show bone lesions with scores over 3. (**d**) Quantification for (**c**), ****P* < 0.001. **e.** Measurement of body weight after LLC inoculation in mice at the

indicated timepoints and treatment groups. Sample sizes are as follows: *n* = 6 vehicle-treated WT mice, *n* = 6 DMXAA-treated WT mice, *n* = 5 vehicle-treated STING$^{gt/gt}$ mice, and *n* = 5 DMXAA-treated STING$^{gt/gt}$ mice. Data represent the mean ± SEM, repeated-measures two-way ANOVA with Bonferroni's *post-hoc* test. Source data are provided as a Source Data file.

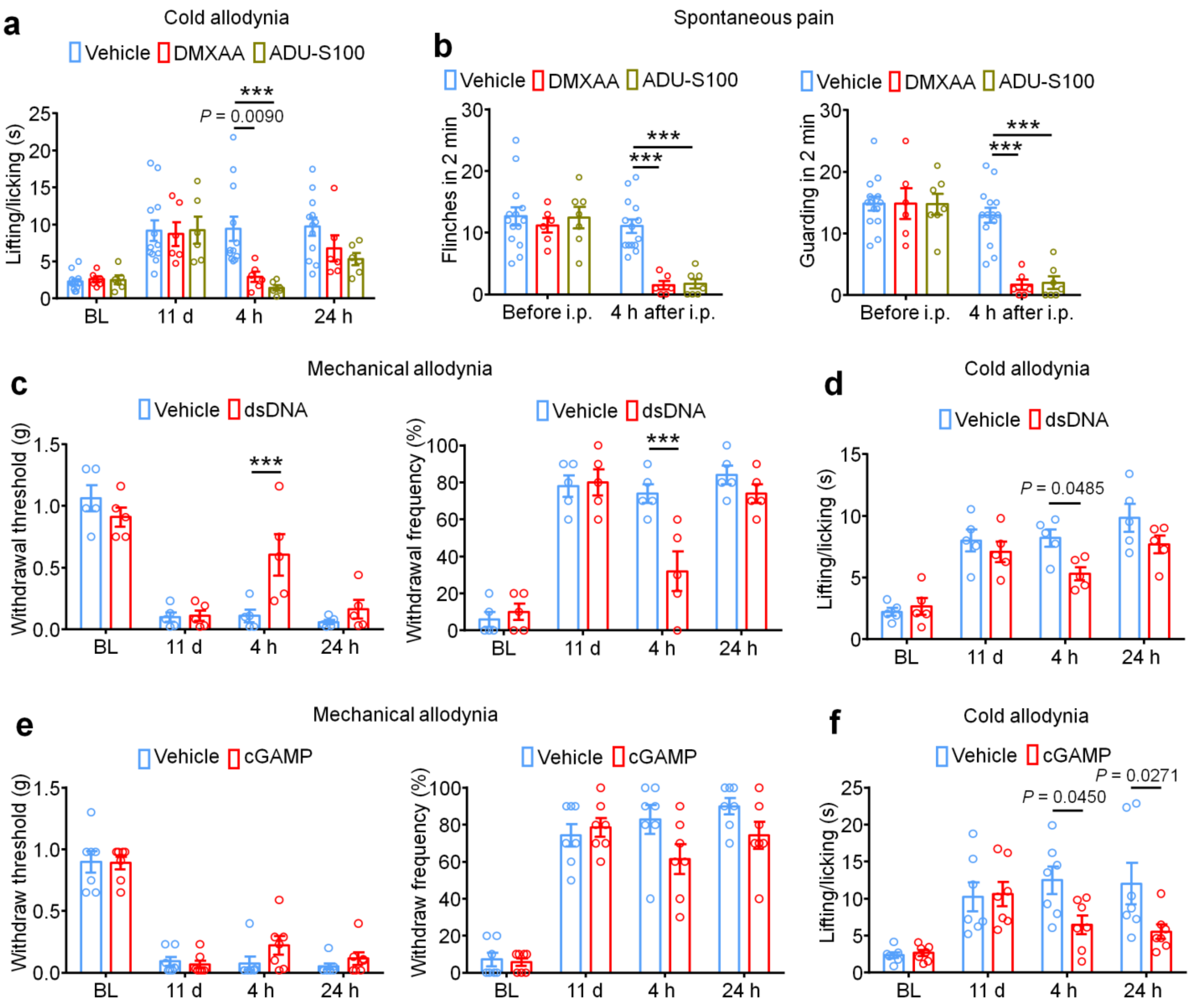

Supplementary Fig. 5. Effects of DMXAA, ADU-S100, and natural STING activators dsDNA and cGAMP on bone cancer pain.

a. Measurement of cold allodynia 4h or 24h after a single i.p. injection of vehicle, DMXAA (20 mg/kg), or ADU-S100 (20 mg/kg) on d11 after LLC inoculation (*n* = 12 vehicle-treated mice; *n* = 6 DMXAA-treated mice, and *n* = 6 ADU-S100-treated mice, pooled from two independent experiments), ***$P < 0.001$. **b**. Quantification of spontaneous pain behaviors, as indicated from flinching (left) or guarding (right) 4h after a single i.p. injection of DMXAA or ADU-S100 at d11 post-LLC (*n* = 14 vehicle-treated mice, *n* = 6 DMXAA-treated mice, and *n* = 7 ADU-S100-treated mice), ***$P < 0.001$. **c-d.** Measurement of cancer-induced mechanical allodynia (**c**) at d11 post LLC inoculation, as determined by withdrawal threshold (**c**, left) or withdrawal frequency (**c**, right) or cold allodynia (**d**) as determined by the acetone test before or 4h or 24h after administration of the natural STING

agonist dsDNA (30 µg, i.p., $n = 5$ mice/group), ***$P < 0.001$. **e-f.** Mechanical allodynia measured by von Frey testing (**e**) or cold allodynia measured by acetone testing (**f**) before and 4h or 24h after treatment with vehicle or the natural STING ligand 3’3’-cGAMP (20 mg/kg, i.p.) on d11 after LLC inoculation ($n = 7$ mice/group). Data indicate the mean ± the SEM, repeated-measures two-way ANOVA with Bonferroni’s *post hoc* test. Source data are provided as a Source Data file.

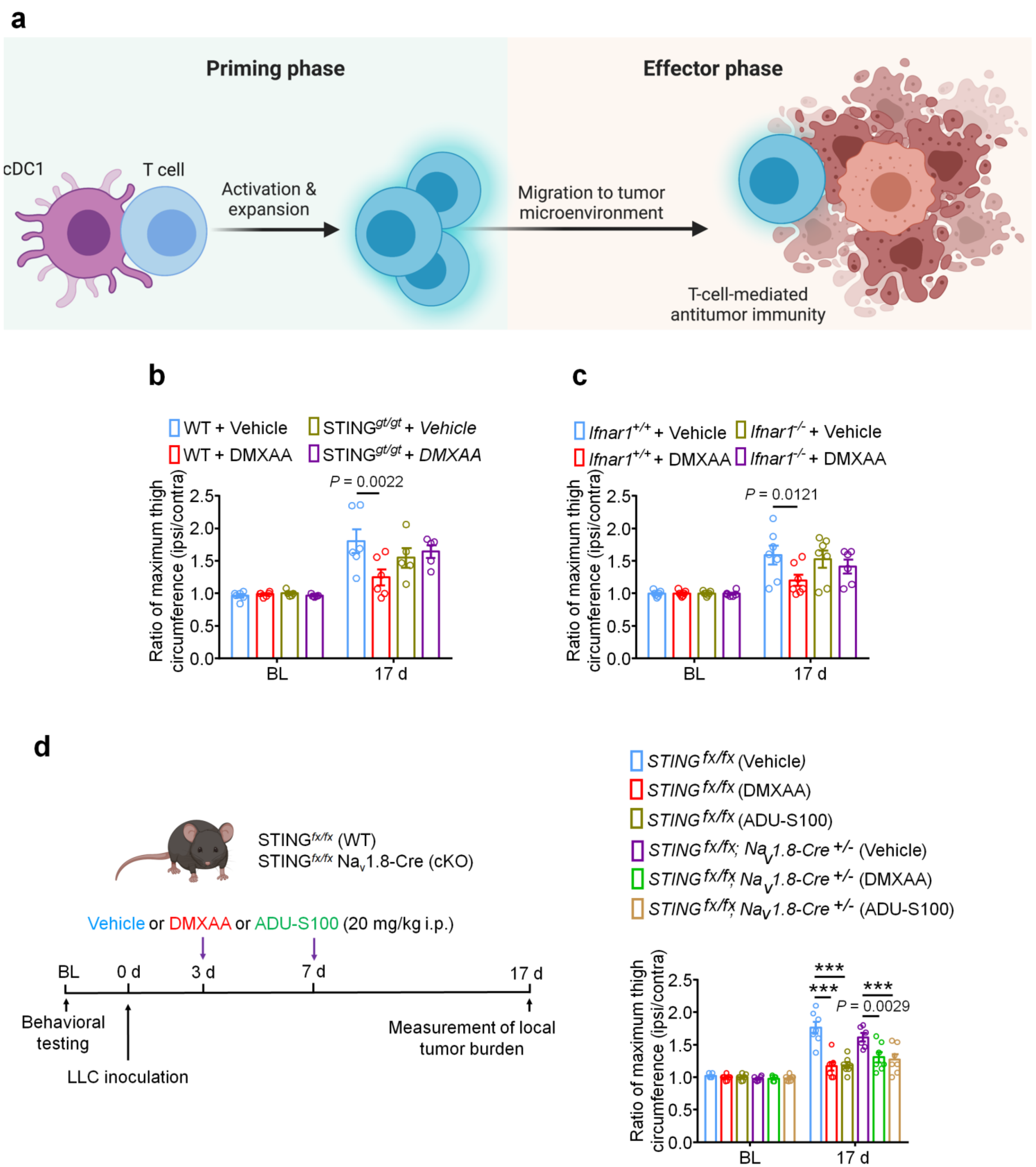

Supplementary Fig. 6. Reduction in local tumor burden by STING agonists requires host-intrinsic STING and IFN-I signaling.

a. Schematic of T cell-mediated anti-tumor immunity. **b.** Ratio of maximum thigh circumference in WT mice or

STING$^{gt/gt}$ mice treated with vehicle or DMXAA (2 x 20 mg/kg, i.p.) on day 17 after LLC implantation, *n* = 6 vehicle-treated WT mice, *n* = 6 DMXAA-treated WT mice, *n* = 5 vehicle-treated STING$^{gt/gt}$ mice, *n* = 5 DMXAA-treated STING$^{gt/gt}$ mice. **c.** Ratio of maximum thigh circumference in WT mice or *Ifnar1*$^{-/-}$ mice treated with vehicle or DMXAA (2 x 20 mg/kg, i.p.) on day 17 after LLC implantation, *n* = 7 vehicle-treated WT mice, *n* = 7 DMXAA-treated WT mice, *n* = 6 vehicle-treated *Ifnar1*$^{-/-}$ mice, *n* = 6 DMXAA-treated *Ifnar1*$^{-/-}$ mice. **d.** Ratio of maximum thigh circumference after DMXAA and ADU-S100 treatment (at d3 and d7, i.p.) in *STING*$^{fx/fx}$; Na_V1.8-Cre (STING-cKO) mice or *STING*$^{fx/fx}$ (WT) mice. Left, schematic of experimental design. Notably, DMXAA and ADU-S100 attenuated tumor burden at d17 in STING-cKO mice, ***$P < 0.001$. Sample sizes are as follows: *n* = 7 vehicle-treated WT mice, *n* = 7 DMXAA-treated WT mice, *n* = 7 ADU-S100-treated WT mice, *n* = 6 vehicle-treated STING-cKO mice, *n* = 7 DMXAA-treated STING-cKO mice, and *n* = 7 ADU-S100-treated STING cKO mice. Data indicate the mean ± SEM, repeated-measures two-way ANOVA with Bonferroni's *post-hoc* test (**b-d**). Source data are provided as a Source Data file.

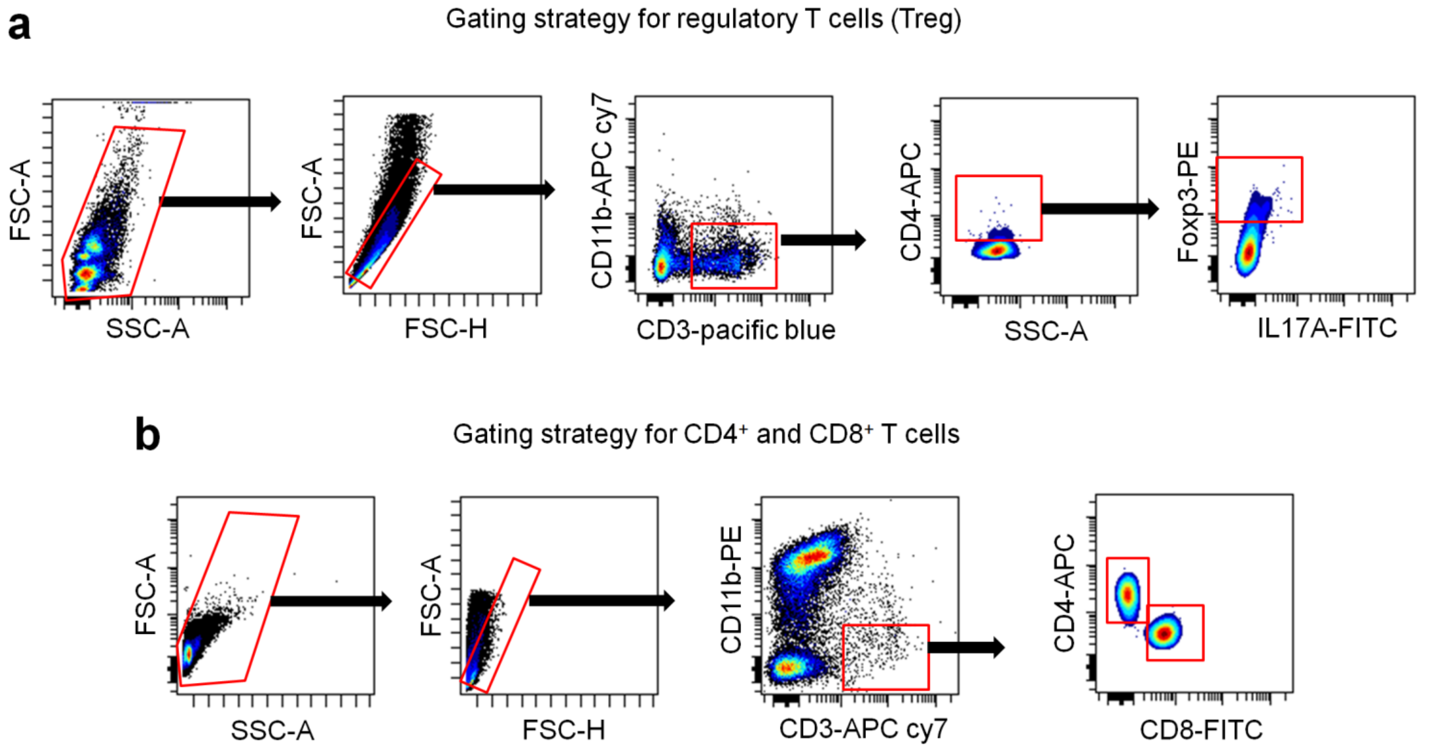

Supplementary Fig. 7. Gating strategy for tumor-infiltrating lymphocytes present in the bone marrow tumor microenvironment. Bone marrow was isolated from the ipsilateral femur of tumor-bearing mice, followed by FACS analysis to quantify the proportion of regulatory T cells (Treg) and $CD4^+$ or $CD8^+$ tumor-infiltrating lymphocytes. Forward scatter (FSC) and side scatter (SSC) were used to identify single cells. **a.** Treg were gated as: single cells → $CD3^+$/$CD11b^-$→ $CD4^+$ → $Foxp3^+$/$IL\text{-}17A^-$. **b.** $CD4^+$ T cells were gated as: single cells → $CD3^+$/$CD11b^-$ → $CD4^+$/$CD8^-$. $CD8^+$ T cells were gated as: single cells → $CD3^+$/$CD11b^-$ → $CD4^-$/$CD8^+$. This gating strategy is related to the data provided in Figure 6**f-g.**

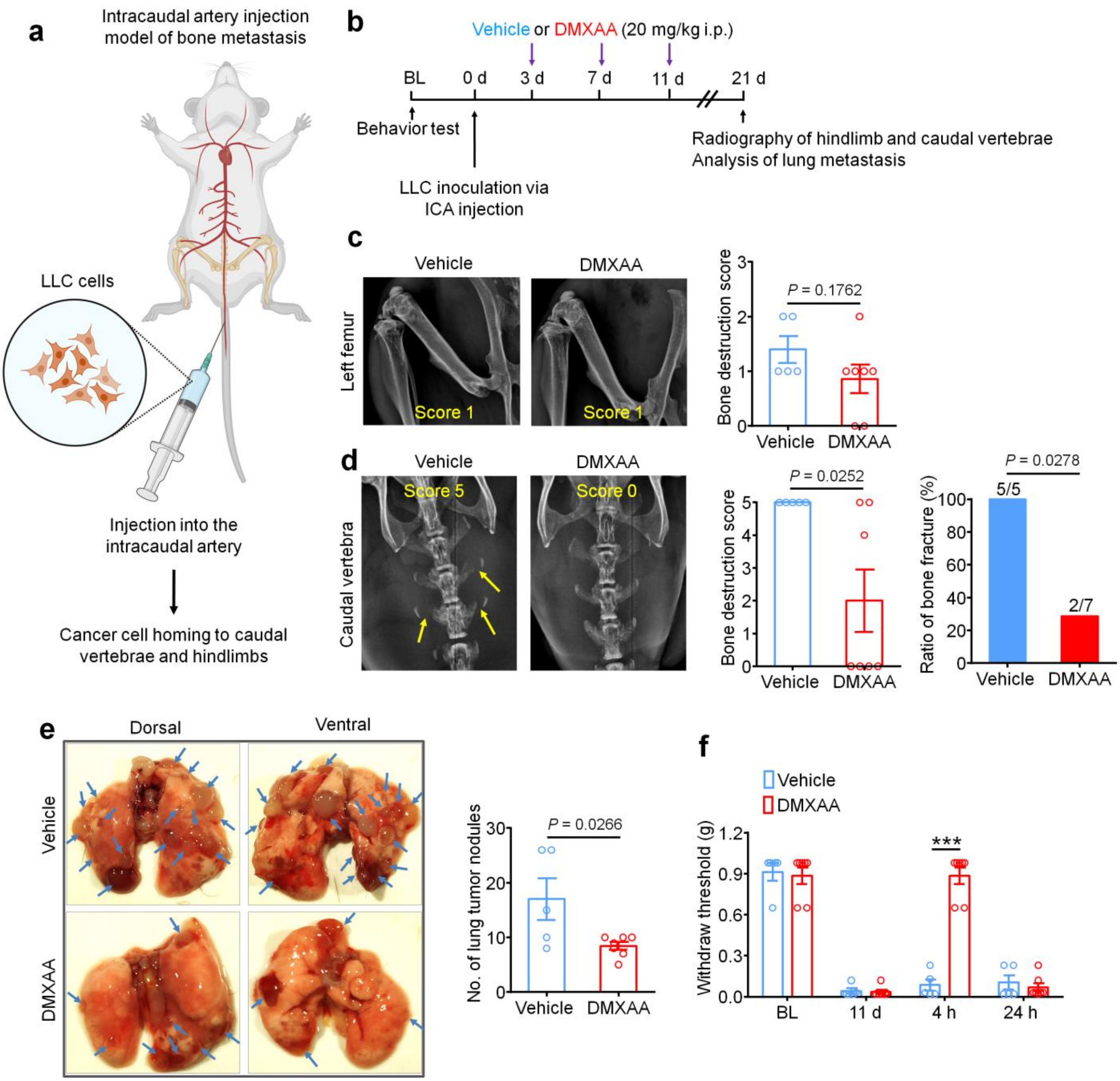

Supplementary Fig. 8. The bone-protective and analgesic effects of STING agonist in a bone metastasis model.

a. Schematic of intra-caudal injection of LLC cells. **b.** Schematic of experimental design. **c-d.** Radiographical analysis of femur (**c**) and caudal vertebrae (**d**) bone destruction in mice administered vehicle or DMXAA (3 x 20 mg/kg, i.p) at d21 post LLC inoculation. Left, representative X-ray images. Bone destruction score is labeled on each photo and arrows indicate bone destruction sites. Right, quantification for bone destruction score and the proportion of mice with caudal vertebrae fracture in both groups. **e**. Image analysis of lung tumor nodules in mice with each indicated treatment on d21 after LLC injection. Left, representative dorsal and ventral murine lung

images, with arrows showing metastatic tumor nodules. Right, quantification of lung tumor nodules. **f**. Paw withdrawal threshold in mice treated with vehicle or DMXAA at d11 post LLC inoculation, ***$P < 0.001$. Sample sizes: n = 5 vehicle-treated mice and n = 7 mice DMXAA-treated mice. Data are Mean ± SEM, two-tailed Student's t-test (**c, d, e**); two-sided Fisher's exact test (**d**); repeated-measures two-way ANOVA with Bonferroni's *post hoc* test (**f**). Source data are provided as a Source Data file.

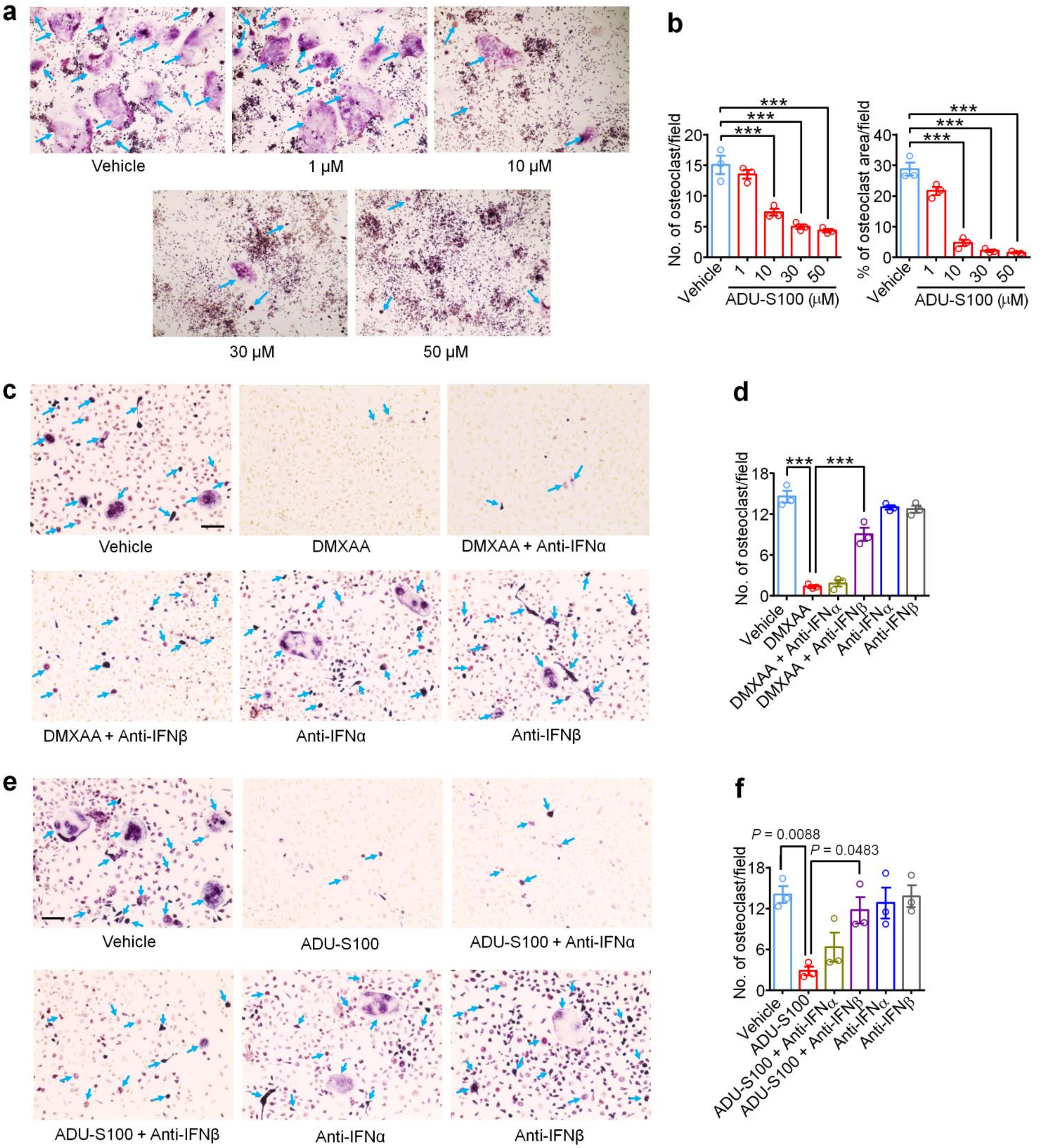

Supplementary Fig. 9. STING agonists suppress osteoclastogenesis via IFN-α and IFN-β.

a-b. TRAP staining from RAW264.7 cells after treatment of ADU-S100**. (a)** Representative images of TRAP staining. (**b**) Quantification of (**a**). n = 3 biologically independent experimental replicates, $***P < 0.001$. **c-f.**

TRAP staining after treatment of DMXAA **(c, d)** and ADU-S100 **(e, f).** (**c, e**) Representative images of TRAP staining to identify *in vitro* BMDM-derived osteoclasts following differentiation with RANKL (35 ng/ml) and M-CSF (20 ng/ml), together with treatment of DMXAA (30 µM) and/or anti-IFN-α antibody (600 ng/ml) or anti-IFN-β antibody (600 ng/ml) (**c**) or treatment of ADU-S100 (30 µM) and/or anti-IFN-α antibody (600 ng/ml) or anti-IFN-β antibody (600 ng/ml) (**e**), Scale bar, 100 µm. (**d**, **f**) Quantification for (**c**) and (**e**). $n = 3$ biologically independent experimental replicates, $^{***}P < 0.001$. Data displayed represent the mean ± SEM, one-way ANOVA with Bonferroni's *post-hoc* test. Source data are provided as a Source Data file.

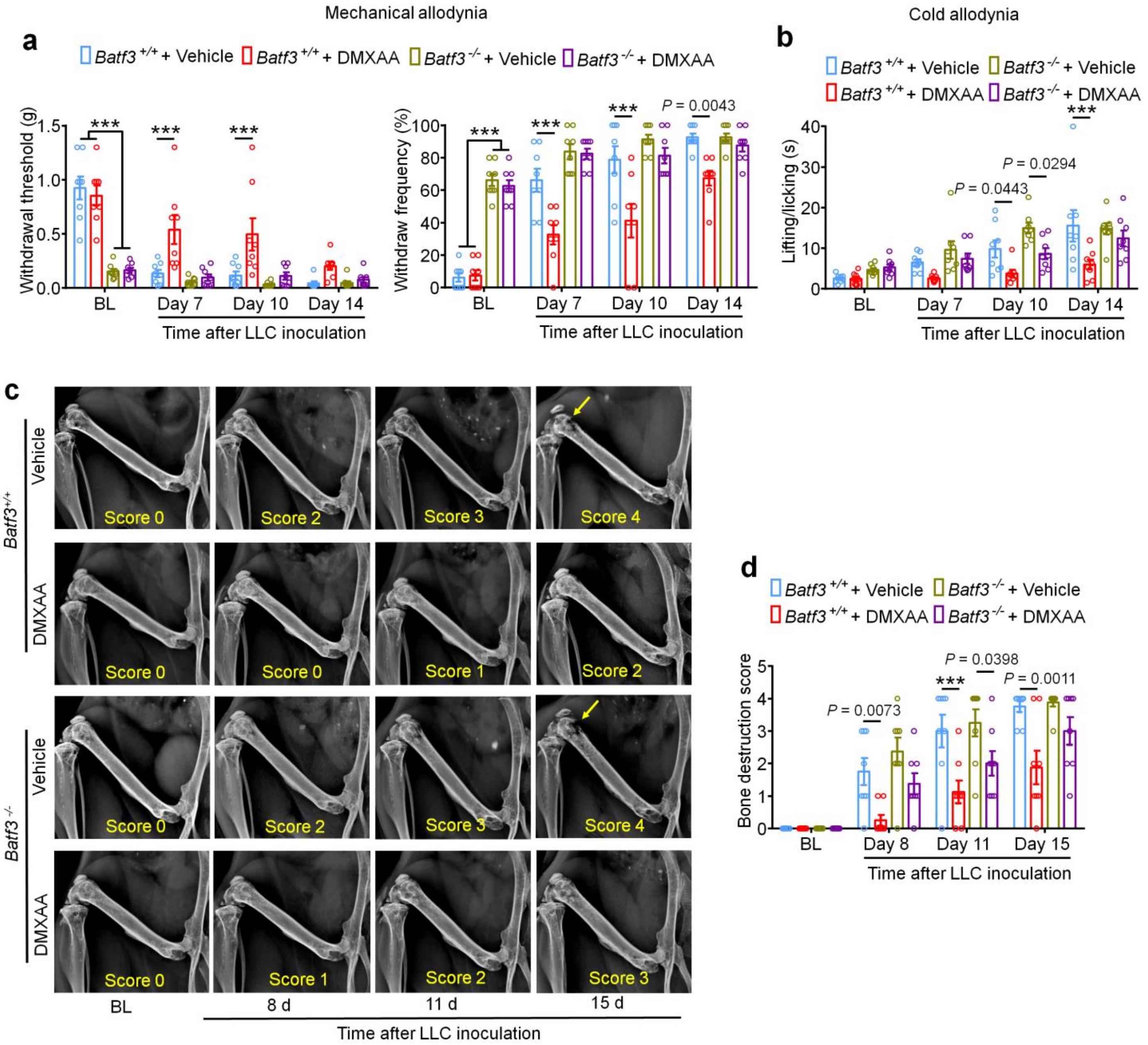

Supplementary Fig. 10. The protective effects of STING agonists remain largely intact until late stages in *Batf3*$^{-/-}$ mice

a. Mechanical allodynia from von Frey test in *Batf3*$^{+/+}$ *or Batf3*$^{-/-}$ mice treated with vehicle or DMXAA (2 x 20 mg/kg, i.p.) on baseline (BL), day 7, 10 and 14 after tumor inoculation (*n* = 8 mice/group), ****P* < 0.001. Left, withdrawal threshold. Right, withdrawal frequency. **b.** Cold allodynia from acetone test in *Batf3*$^{+/+}$ *or Batf3*$^{-/-}$ mice with indicated therapy (*n* = 8 mice/group) ****P* < 0.001. **c-d.** Radiographical analysis of bone destruction in *Batf3*$^{+/+}$ *or Batf3*$^{-/-}$ mice administered vehicle or DMXAA, measured at BL, d8, d11 and d15 post LLC inoculation. (**c**) Representative X-ray images. Bone destruction score is labeled on the bottom of each photo and

arrow indicates bone destruction score more than 3. (**d**) Quantification for (**c**) (n = 8 mice/group) ***$P < 0.001$. Data are Mean ± SEM, repeated-measures two-way ANOVA with Bonferroni's *post hoc* test. Source data are provided as a Source Data file.
